# Quercetin and its metabolite isorhamnetin promote glucose uptake through different signalling pathways in myotubes

**DOI:** 10.1038/s41598-019-38711-7

**Published:** 2019-02-25

**Authors:** Hao Jiang, Yoko Yamashita, Asuka Nakamura, Kevin Croft, Hitoshi Ashida

**Affiliations:** 1 Department of Agrobioscience, Graduate School of Agricultural Science, Kobe University, Kobe, Hyogo 657-8501 Japan; 2 School of Biomedical Science, The University of Western Australia, Perth, WA 6009 Australia

## Abstract

Quercetin and its metabolite isorhamnetin elicit various beneficial effects on human health. However, their bioavailability is low. In this study, we investigated whether low concentrations in the physiological range could promote glucose uptake in L6 myotubes, as well as the underlying molecular mechanisms. We found that 0.1 nM and 1 nM quercetin or 1 nM isorhamnetin significantly increased glucose uptake via translocation of glucose transporter type 4 (GLUT4) to the plasma membrane of L6 myotubes. Quercetin principally activated the CaMKKβ/AMPK signalling pathway at these concentrations, but also activated IRS1/PI3K/Akt signalling at 10 nM. In contrast, 1 nM and 10 nM isorhamnetin principally activated the JAK/STAT pathway. Treatment with siAMPKα and siJAK2 abolished quercetin- and isorhamnetin-induced GLUT4 translocation, respectively. However, treatment with siJAK3 did not affect isorhamnetin-induced GLUT4 translocation, indicating that isorhamnetin induced GLUT4 translocation mainly through JAK2, but not JAK3, signalling. Thus, quercetin preferably activated the AMPK pathway and, accordingly, stimulated IRS1/PI3K/Akt signalling, while isorhamnetin activated the JAK2/STAT pathway. Furthermore, after oral administration of quercetin glycoside at 10 and 100 mg/kg body weight significantly induced GLUT4 translocation to the plasma membrane of skeletal muscles in mice. In the same animals, plasma concentrations of quercetin aglycone form were 4.95 and 6.80 nM, respectively. In conclusion, at low-concentration ranges, quercetin and isorhamnetin promote glucose uptake by increasing GLUT4 translocation via different signalling pathways in skeletal muscle cells; thus, these compounds may possess beneficial functions for maintaining glucose homeostasis by preventing hyperglycaemia at physiological concentrations.

## Introduction

Diabetes mellitus (DM), an epidemic metabolic disorder, is characterized by hyperglycaemia and hyperinsulinaemia resulting from not only impaired insulin secretion, but also insulin resistance. The prevalence of diabetes is growing considerably: the current number of diabetic patients (285 million) is expected to double by 2035^1^. The disease tends to affect younger individuals as a result of diet, behaviour, and obesity^2^. Chronic diabetes is usually accompanied by serious diabetic complications, such as cardiac dysfunction and paropsia disease^3,4^. Therefore, distinguishing novel way to improve insulin resistance and insulin sensitivity is a priority target for treatment or prevention of DM.

Skeletal muscle exerts profound effects on whole-body glucose homeostasis, especially with regard to regulation of hyperglycaemia in the postprandial state. Glucose transporter type 4 (GLUT4), which is specifically expressed in skeletal muscle and adipose tissue^5,6^, is a determinant of glucose transporter for these tissues. Upon insulin stimulus, GLUT4 rapidly translocates to the cell surface from intracellular storage vesicles, which is involved in the activating various protein kinases, including insulin receptor substrate 1 (IRS1), phosphoinositide 3-kinase (PI3K), and Akt^7,8^. Notably, exercise and energy depletion activate adenosine monophosphate-activated protein kinase (AMPK) and its upstream kinases, such as Ca^2+^/calmodulin-dependent kinase kinase (CaMKK) and liver kinase B1 (LKB1), to promote GLUT4 translocation and glucose uptake^9,10^. In the last two decades, Janus kinase 2 (JAK2) and Janus kinase 3 (JAK3) have attracted considerable interest in the context of energy metabolism^11^. Activated JAK2 and JAK3 alter intracellular signalling to result in the activation of signal transducers and transcriptional activators, such as STAT1, STAT3, and STAT5, that participate in multiple biological responses, including tissue homoeostasis, apoptosis, and oncogenesis^12,13^. In addition, activation of the JAK3/STAT3 signalling pathway is involved in glucose uptake in skeletal muscle cells^11^.

Numerous studies have asserted that flavonoids promote translocation of GLUT4 by different signalling pathways in various tissues and cells. For example, flavonoids from propolis extract improve glucose uptake by promoting GLUT4 translocation through both PI3K- and AMPK-dependent pathways in skeletal muscle^14^; whereas, epigallocatechin gallate induces GLUT4 translocation in skeletal muscle through insulin signalling pathways^15^, and procyanidin promotes translocation of GLUT4 in muscle of mice through activation of insulin and AMPK signalling pathways^16^. Quercetin (3,3′,4′,5,7-pentahydroxy flavone) and isorhamnetin (3′-O-methyl quercetin) are considered potential therapeutic agents for various diseases, such as obesity and cancer, as they modulate metabolism, regulate DNA transcription, and activate apoptosis^17–20^. In a previous study, quercetin at 50 mg/kg body weight ameliorated oxidative stress, inflammation, and apoptosis in streptozotocin-nicotinamide-induced diabetic male rats^21^. However, it is important to note that quercetin is poorly absorbed from the intestine. Hence, extensive knowledge of physiological concentrations of quercetin and isorhamnetin are essential for establishing their effects. The absorption rate of quercetin is reportedly 9–20% in humans^22–24^, and basal concentrations of quercetin in the blood range from 300 to 750 nM after consumption of 80–100 mg of quercetin equivalent in humans^24–26^. Furthermore, physiological concentrations of quercetin in tissues are much more important than their plasma concentrations. In rats and mice, physiological concentrations of quercetin in muscle ranged from 0.1 nM to 163 nM^24,25^. In Caco-2 cells, absorption of quercetin was reported to be at the nM level^27^. It is, therefore, necessary to clarify the functions of quercetin and its metabolite isorhamnetin and their underlying mechanism within a physiological concentration range.

In the present study, we investigated whether the mechanism underlying the antidiabetic properties of quercetin and isorhamnetin at a physiological concentration range (nM level) involved promotion of glucose uptake in differentiated L6 myotube cells. We found that 0.1 nM and 1 nM quercetin or 1 nM isorhamnetin significantly enhanced glucose uptake and was accompanied by increased GLUT4 translocation to the plasma membrane of L6 cells by different signalling pathways. Furthermore, we confirmed that after oral administration of quercetin glycoside significant induced GLUT4 translocation to the plasma membrane of skeletal muscles in ICR mice.

## Results

### Quercetin and isorhamnetin promoted glucose uptake in L6 myotubes

**Figure 1:**
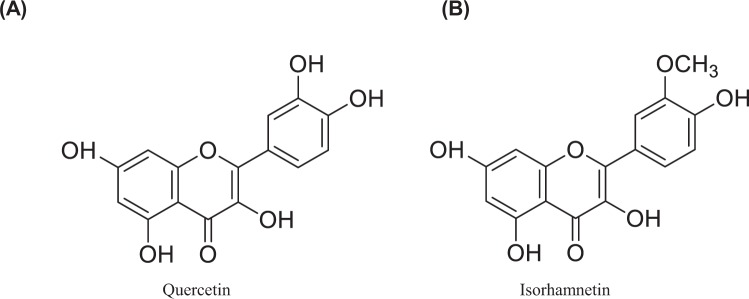
The chemical structures of quercetin and isorhamnetin.

**Figure 2:**
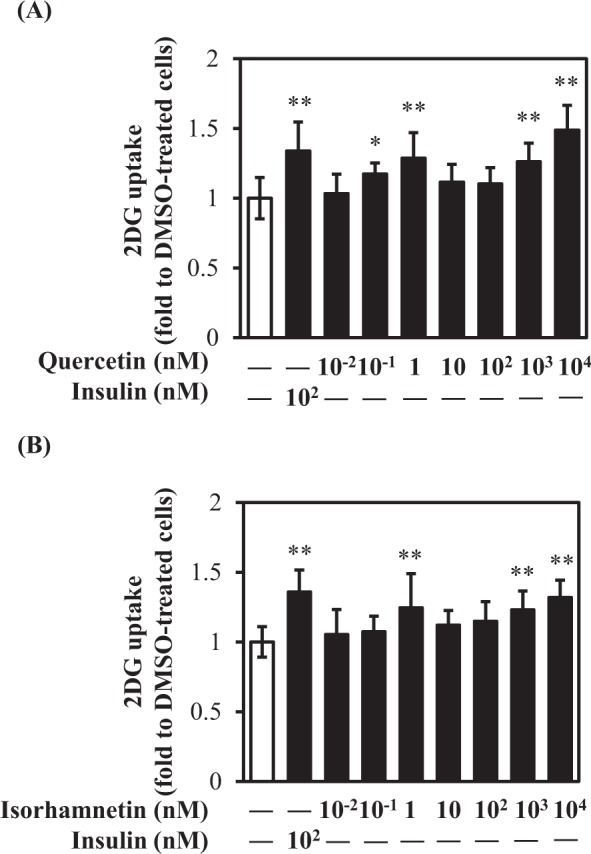
Effect of quercetin and isorhamnetin on glucose uptake in L6 myotubes. Differentiated L6 myotubes were treated with quercetin (**A**) and isorhamnetin (**B**) at the indicated concentrations for 4 h. Glucose uptake was determined using an enzymatic 2DG uptake assay. Data shown represent mean ± SD (n = 3). * and ** indicate significant differences from control cells by Dunnett’s multiple comparison test (*p < 0.05 and **p < 0.01, respectively).

We first investigated whether quercetin or isorhamnetin (chemical structures are shown in Fig. 1) could promote glucose uptake at 0.01–10^4^ nM in L6 myotubes. The results showed that quercetin and isorhamnetin increased glucose uptake in a dose-dependent manner from 0.01 nM to 1 nM (Fig. 2), compared with vehicle (dimethyl sulfoxide, DMSO) treatment. A significant increase was observed at 0.1 nM and 1 nM quercetin, and 1 nM isorhamnetin. At 10 nM, both compounds decreased glucose uptake. However, they again increased glucose uptake at a higher concentration range in a dose-dependent manner. At 1 μM and 10 μM, both compounds elicited a significant increase. From these results, quercetin and isorhamnetin facilitated a biphasic increase in glucose uptake in L6 myotubes.

### Quercetin and isorhamnetin promote GLUT4 translocation

**Figure 3:**
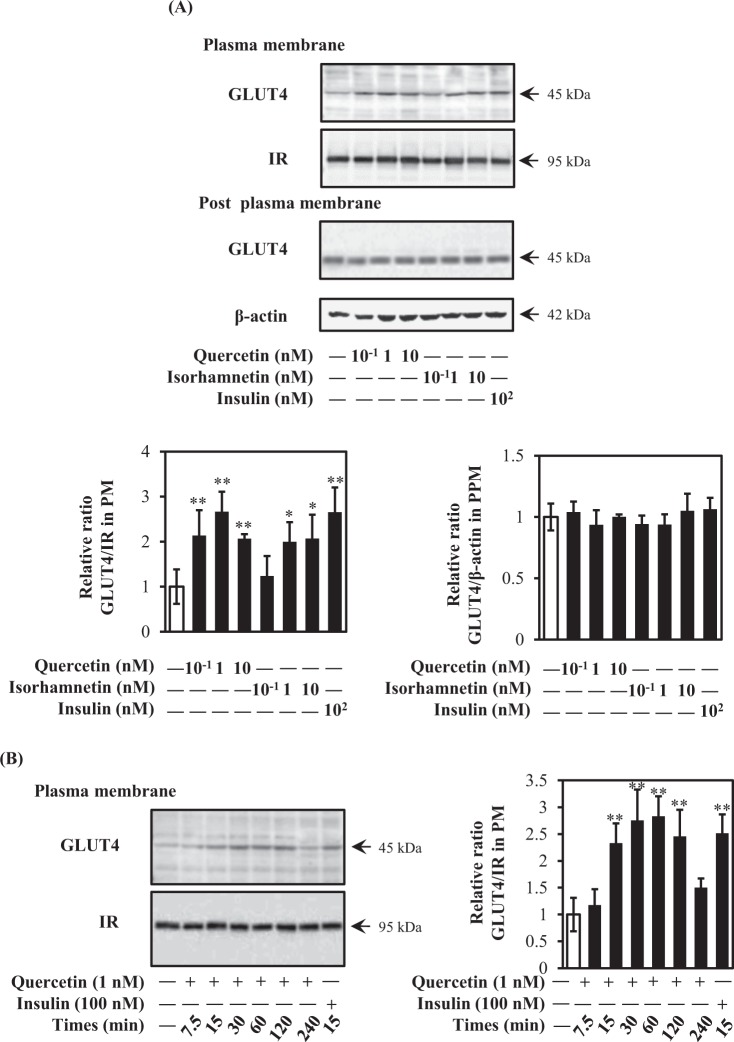
Effect of quercetin and isorhamnetin on GLUT4 translocation in L6 myotubes. (**A**) Differentiated L6 myotubes were treated with quercetin and isorhamnetin at the indicated concentrations for 15 min. Plasma membrane fractions were prepared and subjected to analysis of GLUT4 translocation by western blotting. Arrow showed the target protein bands. Original blots are shown in Supplementary Fig. 4. Representative data are shown from independent triplicate analyses. Band density was measured and represented as the ratio of GLUT4 to IRβ. (**B**) Differentiated L6 myotubes were treated with 1 nM quercetin for the indicated times (0–240 min). Plasma membrane fractions were prepared and subjected to analysis of GLUT4 translocation by western blotting. Arrow showed the target protein bands. Original blots are shown in Supplementary Fig. 4. Representative data are shown from independent triplicate analyses. Band density was measured and represented as the ratio of GLUT4 to IRβ. Data shown represent mean ± SD (n = 3). * and ** indicate significant differences from control cells by Dunnett’s multiple comparison test (*p < 0.05 and **p < 0.01, respectively).

Because GLUT4 incorporates glucose into skeletal muscle cells after translocation from intracellular storage sites to the plasma membrane, we next investigated GLUT4 translocation after treatment of L6 myotubes with quercetin or isorhamnetin at 0.1–10 nM for 15 min. With the exception of 0.1 nM isorhamnetin, both compounds significantly increased GLUT4 translocation to almost the same extent as the 100-nM insulin positive control (Fig. 3A). However, the expression level of GLUT4 remained unchanged (Fig. 3A). When time-dependent changes in GLUT4 translocation were monitored for 240 min, quercetin-induced translocation exhibited a bell-shaped curve: a significant increase appeared at 15 min and then the translocation level plateaued from 30 to 60 min, decreased from 60 min, and was restored by 240 min (Fig. 3B). From these results, quercetin and isorhamnetin at a physiological concentration range increased glucose uptake by promoting GLUT4 translocation to the plasma membrane without altering GLUT4 expression levels in L6 myotubes.

### Quercetin activated both insulin- and AMPK-dependent pathways in L6 myotubes

**Figure 4:**
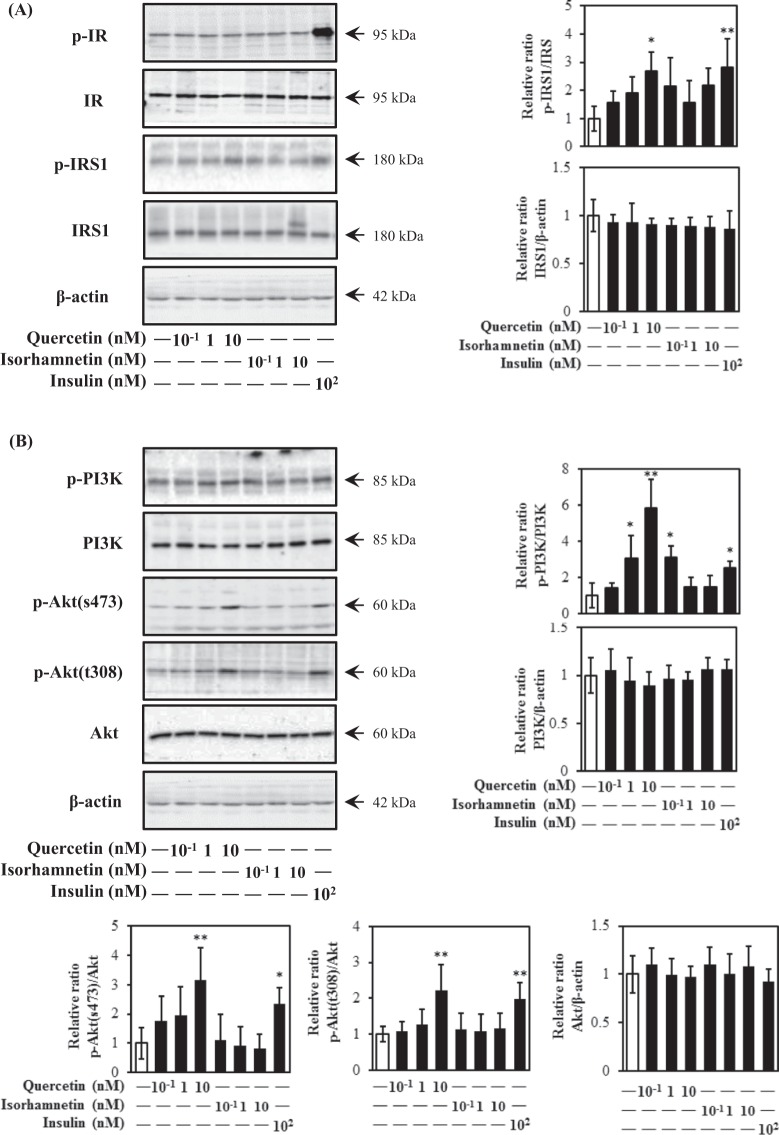
Effect of quercetin and isorhamnetin on the insulin signalling pathway in L6 myotubes. Differentiated L6 myotubes were treated with quercetin and isorhamnetin at the indicated concentrations for 15 min. Cell lysates were prepared and subjected to analysis of phosphorylation and expression of proteins in the insulin signalling pathway by western blotting. Arrow showed the target protein bands. Original blots are shown in Supplementary Fig. 5. Representative data are shown from independent triplicate analyses. (**A**) Band density was measured and represented as the ratio of p-IR to IR or p-IRS1 to IRS1. (**B**) Band density was measured and represented as the ratio of p-PI3K to PI3K or p-Akt to Akt. Data shown represent mean ± SD (n = 3). * and ** indicate significant differences from control cells by Dunnett’s multiple comparison test (*p < 0.05 and **p < 0.01, respectively).

Translocation of GLUT4 is mainly regulated by insulin- and AMPK-dependent pathways. To explore molecular mechanisms of quercetin- and isorhamnetin-induced GLUT4 translocation, involvement of these pathways was examined. Both quercetin and isorhamnetin failed to activate phosphorylation of insulin receptors (IRs) at all concentrations tested (Fig. 4A). Interestingly, quercetin, but not isorhamnetin, dose-dependently promoted phosphorylation of IRS1, a downstream target of IR. In addition, quercetin significantly increased phosphorylation of PI3K, which is downstream of IRS1 (Fig. 4B). Surprisingly, 0.1 nM isorhamnetin also increased PI3K phosphorylation without affecting IRS1 phosphorylation. Regarding Akt, 10 nM quercetin increased Akt phosphorylation at Thr308 and Ser473 as the same extent as insulin, while isorhamnetin did not affect Akt phosphorylation. However, quercetin and isorhamnetin did not affect expression level of these proteins. At higher concentrations, quercetin and isorhamnetin significantly increased Akt phosphorylation at Ser473 (Supplementary Fig. 1).

**Figure 5:**
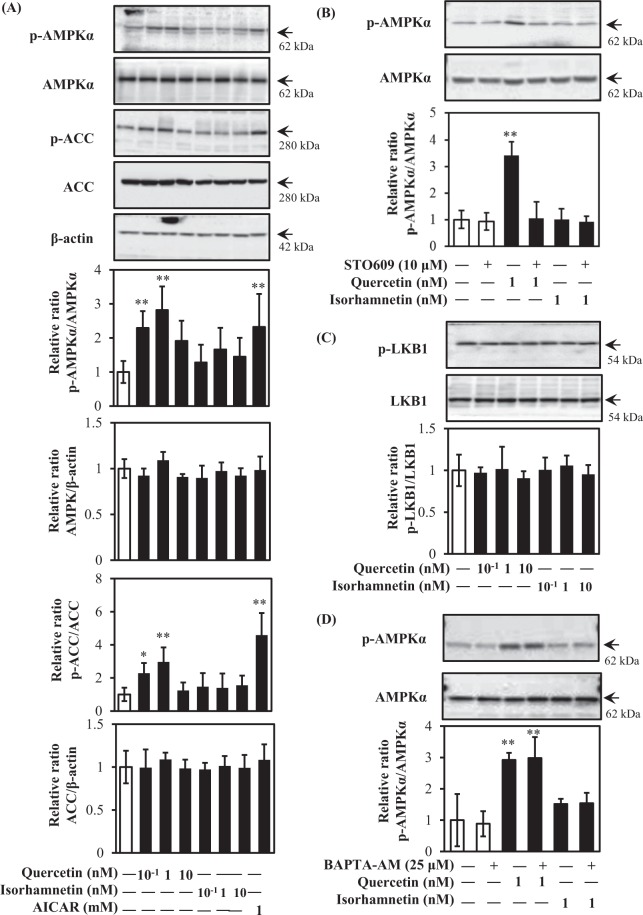
Effect of quercetin and isorhamnetin on the AMPK signalling pathway in L6 myotubes. Differentiated L6 myotubes were treated with quercetin and isorhamnetin at the indicated concentrations for 15 min in the absence or presence of STO-609 or BADPA-AM, respectively. Cell lysates were prepared and subjected to analysis of phosphorylation and expression of proteins in the AMPK signalling pathway by western blotting. Arrow showed the target protein bands. Original blots are shown in Supplementary Fig. 6. Representative data are shown from independent triplicate analyses. (**A**) Differentiated L6 myotubes were treated with quercetin and isorhamnetin at the indicated concentrations for 15 min. Band density was measured and represented as the ratio of p-AMPK to AMPK, or p-ACC to ACC. (**B**) Differentiated L6 myotubes were treated with quercetin and isorhamnetin at the indicated concentrations for 15 min in the presence of STO-609. Band density was measured and represented as the ratio of p-AMPK to AMPK. (**C**) Differentiated L6 myotubes were treated with quercetin and isorhamnetin at the indicated concentrations for 15 min. Band density was measured and represented as the ratio of p-LKB1 to LKB1. (**D**) Differentiated L6 myotubes were treated with quercetin and isorhamnetin at the indicated concentrations for 15 min in the presence of BADPA-AM. Band density was measured and represented as the ratio of p-AMPK to AMPK. Data shown represent mean ± SD (n = 3). * and ** indicate significant differences from control cells by Dunnett’s multiple comparison test (*p < 0.05 and **p < 0.01, respectively).

AMPK is recognized as a metabolic sensor for the prevention of obesity and type 2 diabetes^6,9^. GLUT4 translocation is triggered by the activation of AMPK as an insulin-independent mechanism^10^. As shown in Fig. 5, 0.1 nM and 1 nM quercetin, but not isorhamnetin, promoted AMPK phosphorylation similar to the positive control 5-aminoimidazole-4-carboxyamide ribonucleoside (AICAR) (Fig. 5A). At higher concentrations (1 μM and 10 μM), both compounds induced phosphorylation of AMPK (Supplementary Fig. 2). Phosphorylation of acetyl-CoA carboxylase (ACC), a downstream target of AMPK, also showed the same trend as phosphorylation of AMPK, i.e., phosphorylation was increased by treatment with 0.1 nM or 1 nM quercetin. To obtain further information about factors upstream of AMPK, we examined the involvement of LKB1, CaMKKβ, and intracellular-free calcium (Ca^2+^). As shown in Fig. 5B, the CaMKKβ inhibitor STO-609 abolished quercetin-induced AMPK phosphorylation. In contrast, neither quercetin nor isorhamnetin increased LKB1 phosphorylation (Fig. 5C). The intracellular Ca^2+^ chelator 1,2-Bis (2-aminophenoxy) ethane-*N,N,N′,N′*-tetraacetic acid tetrakis (acetoxymethyl ester) (BAPTA-AM) did not affect quercetin-induced AMPK phosphorylation (Fig. 5D). These results indicated that quercetin principally activated the CaMKKβ/AMPK signalling pathway at 0.1 and 1 nM, and activated the IRS1/PI3K/Akt signalling pathway at 10 nM to induce GLUT4 translocation in L6 cells.

### Isorhamnetin activated a JAK/STAT-dependent pathway in L6 myotubes

**Figure 6:**
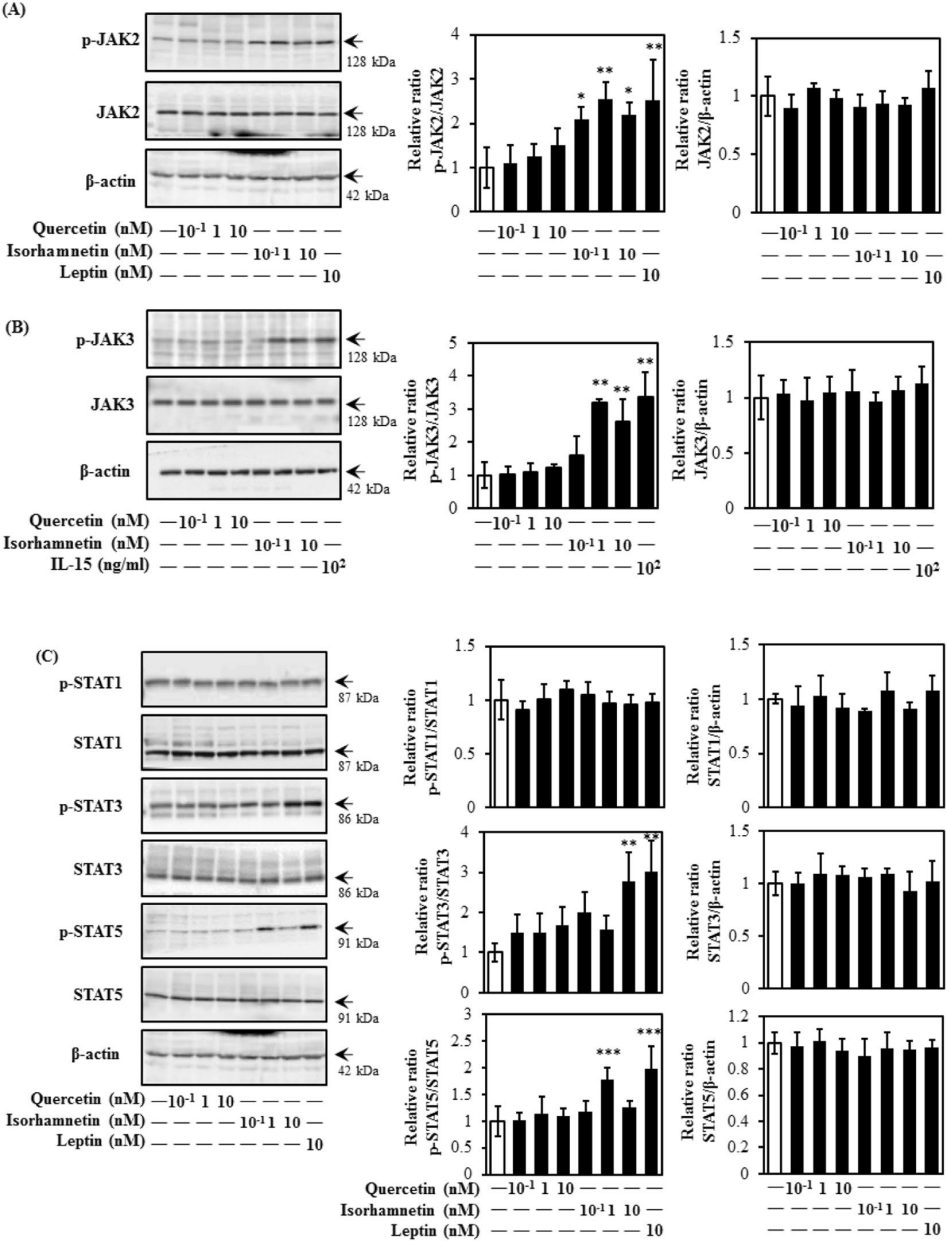
Effect of quercetin and isorhamnetin on the JAK/STAT signalling pathway in L6 myotubes. Differentiated L6 myotubes were treated with quercetin and isorhamnetin at the indicated concentrations for 15 min. Cell lysates were prepared and subjected to analysis of phosphorylation and expression of proteins in the JAK/STAT signalling pathway by western blotting. Arrow showed the target protein bands. Original blots are shown in Supplementary Fig. 7. Representative data are shown from independent triplicate analyses. Band density was measured and represented as the ratio of (**A**) p-JAK2 to JAK2, (**B**) p-JAK3 to JAK3, or (**C**) p-STAT1 to STAT1, p-STAT3 to STAT3, or p-STAT5 to STAT5. Data shown represent mean ± SD (n = 3). *, **, and *** indicate significant differences from control cells by Dunnett’s multiple comparison test (*p < 0.05, **p < 0.01 and ***p < 0.001, respectively).

The JAK/STAT-pathway is associated with maintaining glucose homeostasis and inducing translocation of GLUT4^11^. As shown in Fig. 6, isorhamnetin, but not quercetin, effectively promoted JAK2 and JAK3 phosphorylation at a physiological concentration range (Fig. 6A,B). Concurrently, phosphorylation of downstream targets STAT3 and STAT5, but not STAT1, were increased by isorhamnetin at 10 nM and 1 nM, respectively (Fig. 6C). As shown in Supplemental Fig. 3, both isorhamnetin and quercetin induced phosphorylation of JAK2 at higher concentrations (1 μM and 10 μM). From these results, isorhamnetin at physiological concentrations induced translocation of GLUT4 through the JAK/STAT pathway.

**Figure 7:**
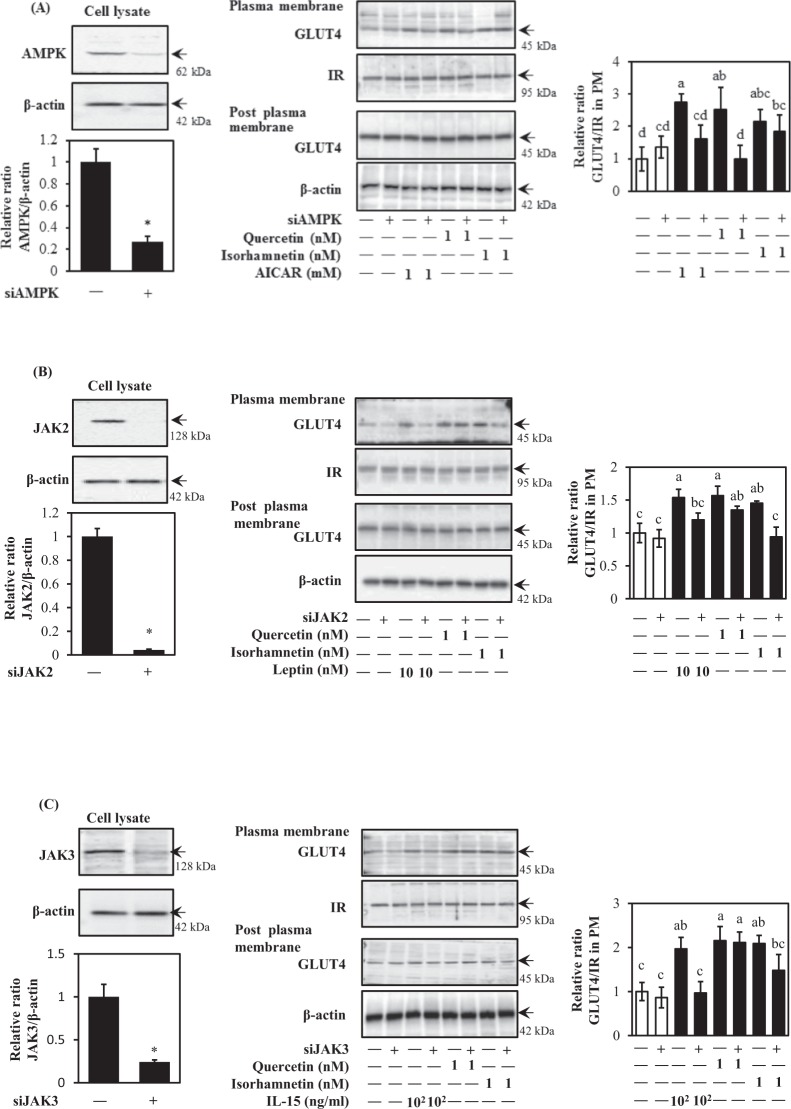
Effect of quercetin and isorhamnetin on GLUT4 translocation via AMPK- and JAK/STAT-dependent pathways. Differentiated L6 myotubes were transfected with siRNAs targeting (**A**) AMPKα, (**B**) JAK2, (**C**) JAK3, or control siRNA and treated with quercetin and isorhamnetin at the indicated concentrations for 15 min. The plasma membrane fraction was prepared and subjected to analysis of GLUT4 translocation by western blotting. Arrow showed the target protein bands. Original blots are shown in Supplementary Fig. 8. Representative data are shown from independent triplicate analyses. Band density was measured and represented as the ratio of GLUT4 to IRβ. Data shown represent mean ± SD (n = 3). * and ** indicate significant differences from control cells by student’s *t*-test (*p < 0.05, and **p < 0.01 respectively). Values with the same letters are not significantly different by Tukey–Kramer multiple comparison test (*p* < 0.05).

To further confirm the roles of AMPKα, JAK2, and JAK3 in quercetin- and isorhamnetin-induced GLUT4 translocation, siRNA was introduced. As shown in Fig. 7A, siRNA for AMPK almost completely abolished quercetin- and AICAR-induced GLUT4 translocation to the control level, but failed to suppress isorhamnetin-induced GLUT4 translocation. In contrast, siRNA for JAK2 abolished isorhamnetin- and leptin-induced GLUT4 translocation without affecting quercetin-induced GLUT4 translocation (Fig. 7B). Isorhamnetin-induced GLUT4 translocation was slightly decreased after treatment with siRNA for JAK3, although this was not significant (Fig. 7C). From these results, we confirmed that quercetin-induced GLUT4 translocation to the cell surface is primarily dependent on the AMPK pathway, while isorhamnetin-induced GLUT4 translocation is mainly due to JAK2, but not JAK3, signalling. These results indicated that AMPKα and JAK2 contributed to the GLUT4-mediated glucose uptake induced in muscle cells by quercetin and isorhamnetin, respectively.

### Enzymatically modified isoquercitrin (EMIQ) promote GLUT4 translocation *in vivo*

**Figure 8:**
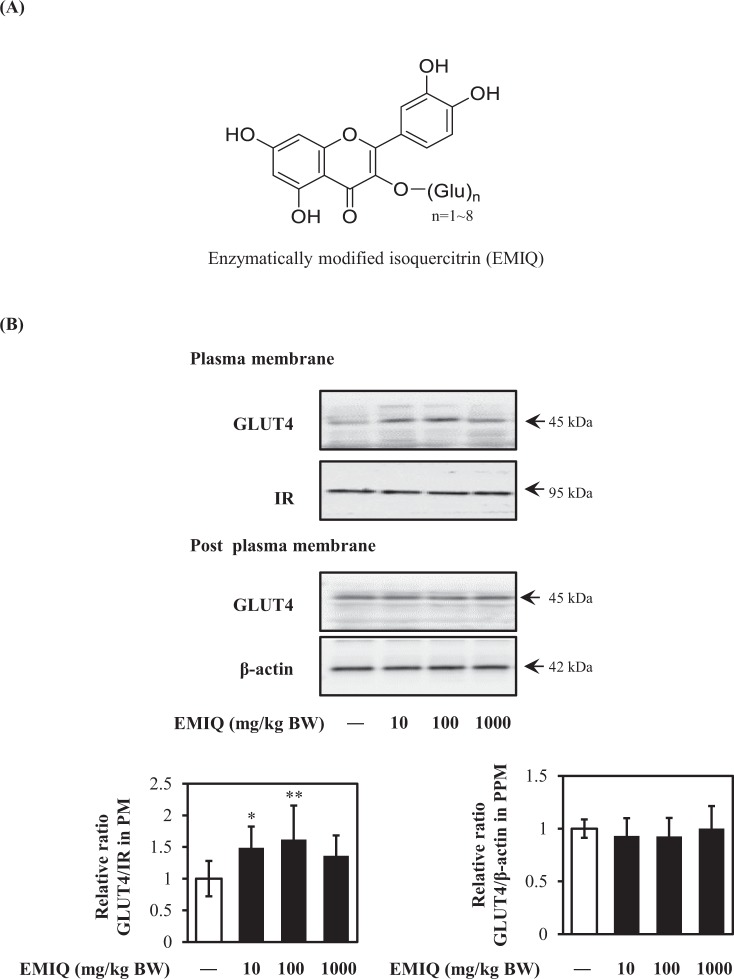
Effect of EMIQ on GLUT4 translocation in mice. (**A**) The chemical structures of EMIQ. (**B**) GLUT4 translocation in mice muscle 90 min after oral administration of EMIQ. Plasma membrane fraction was prepared and subjected to analysis of GLUT4 translocation by western blotting. Arrow showed the target protein bands. Original blots are shown in Supplementary Fig. 9. Each bar graph shows typical result from five animals. The band density was measured and represented as the ratio of GLUT4/IRβ in the plasma membrane fraction or the ratio of GLUT4/β-actin in tissue lysate. Data were shown as the mean ± SE (n = 5). * and ** indicate significant difference from the control group by Dunnett’s multiple comparison test (*p < 0.05 and **p < 0.01).

Quercetin mainly exists in plant and plant-derived foods as its glycoside forms, such as rutin (quercetin-3-O-β-rutinoside) and isoquercitrin (quercetin-3-O-β-glucoside)^28^. Enzymatically modified isoquercitrin (EMIQ, chemical structure is shown in Fig. 8A) is a quercetin derivatives having 1–8 linear glucose moiety at C-3 position of quercetin structure^29^. It was reported that the bioavailability of EMIQ is 17-fold higher than that of quercetin^29^. Thus, EMIQ was orally given to ICR mice at 10, 100 and 1000 mg/kg body weight, and GLUT4 translocation in muscle of mice was determined. As a result, EMIQ at 10 and 100 mg/kg body weight significantly induced GLUT4 translocation to the plasma membrane in skeletal muscles of mice (Fig. 8B).

Furthermore, we quantified the plasma concentration of quercetin and isorhamnetin after administration of EMIQ by high performance liquid chromatography (HPLC). As shown in Table 1, the concentrations of quercetin aglycone in the plasma were 4.95 ± 0.82 nM, 6.80 ± 2.00 nM, and 138.43 ± 45.14 nM, 90 min after oral administration of EMIQ at 10, 100, and 1000 mg/kg body weight, respectively. Meantime, the total (aglycone plus conjugated forms) concentrations of quercetin were 11.69 ± 3.99 nM, 54.89 ± 27.44 nM, and 571.60 ± 225.41 nM. After administration of EMIQ at 1000 mg/kg body weight, isorhamnetin aglycone was detected at 130.74 ± 51.52 nM, though it was not detected in the plasma after administration of EMIQ at 10, and 100 mg/kg body weight. The total concentrations of isorhamnetin were 2.75 ± 2.77 nM, 14.62 ± 4.62 nM, and 351.83 ± 139.88 nM, after administration of EMIQ at 10, 100, and 1000 mg/kg body weight. These results suggested that quercetin at a physiological concentration range induced GLUT4 translocation *in vivo*.

**Table 1:** Concentrations of quercetin and isorhamnetin in plasma of mice after orally administration of EMIQ.

Compounds		EMIQ 10 mg/kg body weight	EMIQ 100 mg/kg body weight	EMIQ 1000 mg/kg body weight
Quercetin	Aglycone form	4.95 ± 0.82	6.80 ± 2.00	138.43 ± 45.14
	Conjugated form	11.69 ± 3.99	54.89 ± 27.44	571.60 ± 225.41
Isorhamnetin	Aglycone form	N.D.	N.D.	130.74 ± 51.52
	Conjugated form	2.75 ± 2.77	14.62 ± 4.62	351.83 ± 139.88

ICR mice were orally administrated EMIQ at 10, 100, or 1000 mg/kg body weight, or water as a vehicle control after 18 h fasting. Blood was collected from a cardiac puncture 90 min after the administration. Plasma was prepared and used for measurement of quercetin and isorhamnetin by HPLC with or without deconjugation.

## Discussion

Prevention of hyperglycaemia is important to reduce the onset of diabetes mellitus. As a result of the drug resistance and toxic side effects associated with current chemotherapy, scientists have recently paid greater attention to food components, especially polyphenols and polyphenol-rich food materials. For example, procyanidin-rich cacao liquor procyanidin extract^5^, glabridin^6^, and epigallocatechin gallate^15^ have been reported to prevent hyperglycaemia. Quercetin is one of the most abundant dietary flavonoids and its average daily consumption is 25–50 mg per day^30^. Quercetin metabolism mainly occurs in the small intestine and liver, where quercetin is biotransformed to isorhamnetin and tamarixetin^31,32^. Quercetin and isorhamnetin have positive impacts on many health functions, including reduced risks of cardiovascular disease, cancer, and obesity^18–21^. Nonetheless, their poor bioavailability^22–24,33^ limits their clinical applications. Considering these concerns, it is imperative to clarify if their beneficial functions occur within a physiological concentration range which can be achieved by dietary intake. In this study, we first observed that physiological concentrations of both quercetin and isorhamnetin promoted glucose uptake and induced GLUT4 translocation to the plasma membrane in rat L6 skeletal muscle cells.

Quercetin and isorhamnetin elicited a biphasic increase in glucose uptake in L6 myotubes. They increased glucose uptake in a dose-dependent manner from 0.01 nM to 1 nM and decreased glucose uptake at 10 nM and 100 nM (Fig. 2). However, they again increased glucose uptake at 1 μM and 10 μM in a dose-dependent manner (Fig. 2). This intriguing complicated biphasic increase in glucose uptake is speculated to involve migration and various non-covalent interactions, including hydrogen bonds, van der Waals forces, electrostatic interactions, and hydrophobic interactions in the solvent^34^. These interactions are involved in complexation of quercetin and isorhamnetin with macromolecules, which affects the solubility and absorption rate of quercetin and isorhamnetin in target tissues^35^. Another possible explanation is that serotonylation of Rab and/or Rho proteins are involved in the underlying mechanism of quercetin- and isorhamnetin-induced glucose uptake. Serotonin reportedly shows a similar biphasic increase trend of glucose uptake by serotonylation of the small GTPase Rab4 in L6 cells^36^. These results may explain, at least in part, the biphasic action of quercetin- and isorhamnetin-induced glucose uptake observed in this study, although further experiments are needed to clarify this unique action.

Although isorhamnetin is a metabolite of quercetin, they have different physiological properties in terms of radical scavenging, enzymatic, and vasodilator activities^32^. In this study, physiological concentrations of quercetin and isorhamnetin promoted GLUT4 translocation to the plasma membrane in L6 myotubes through different mechanisms without altering GLUT4 expression. Quercetin principally activated CaMKKβ/AMPK/ACC signalling at 0.1 nM and 1 nM (Fig. 5). Quercetin- and AICAR-induced glucose uptake (data not shown) and GLUT4 translocation were significantly suppressed by siRNA for AMPK. CaMKKβ belongs to the Ca^2+^/calmodulin-dependent protein kinase family and plays a role in the calcium/calmodulin-dependent kinase cascade. In this study, quercetin activated CaMKKβ phosphorylation without affecting the intracellular calcium concentration, although CaMKKβ is closely linked to Ca2+/calmodulin. Notably, calcium increases glucose uptake by muscle cells via both CaMKKβ/AMPK-dependent and -independent mechanisms^37^. Moreover, androgen, ghrelin, and AMP/ATP are also involved in CaMKKβ phsophorylation^38,39^. Because we did not address the mechanism by which quercetin activated CaMKKβ, further study is needed to clarify this issue.

Isorhamnetin at 1 nM and 10 nM principally activated the JAK/STAT pathway to induce GLUT4 translocation (Fig. 6). The JAK/STAT-pathway is important during carcinogenesis, in particular metastasis^11,12^. Our finding is the first report demonstrating the involvement of JAK2 and STAT3/5 phosphorylation in the promotion of GLUT4 translocation induced by isorhamnetin in skeletal muscle cells (Fig. 7C). This result is at least partially consistent with a previous report showing that IL-15 increased glucose uptake and induced GLUT4 translocation through JAK3/STAT3 signalling^11^. Isorhamnetin at 0.1 nM–10 nM induced JAK2 phosphorylation, while its downstream factors STAT3 and STAT5 were phosphorylated by isorhamnetin at 10 nM and 1 nM, respectively (Fig. 6C). One conceivable reason for this observation is that JAK2 regulates STAT1, STAT3 and STAT5 by different mechanisms, as leptin repressed the JAK2-STAT3/PI3K pathway in a rat model, while growth hormone regulated the phosphorylation of JAK2 and STAT5 in flounder^40,41^. Notably, 0.1 nM isorhamnetin also induced PI3K phosphorylation, but this was not reflected in its downstream targets, such as Akt and GLUT4 translocation (Fig. 4B). One possible explanation involves the SH2B domain, which connects the JAK/STAT and insulin signalling pathways^40,42,43^. Indeed, the SH2B domain is reportedly involved in phosphorylation of JAK2, IRS1, IRS2, and PI3K in response to leptin^41^. In addition, PTP1B, a negative regulator of insulin and JAK/STAT signalling pathways, also has the potential to cause this condition^44^. Fudan-Yueyang *Ganoderma lucidum* extract has been reported to decrease blood glucose level and ameliorate insulin resistance by decreasing PTP1B expression and increasing PI3K phosphorylation^44^.

Furthermore, 10 nM quercetin activated phosphorylation of IRS1, PI3K, and two amino acid residues on Akt (Thr308 and Ser473), suggesting that quercetin acted in the same manner as insulin by completely activating the function of Akt to regulate glucose levels (Fig. 4). IR was not involved in quercetin- and isorhamnetin-induced GLUT4 translocation, although IRS1, PI3K, and Akt phosphorylation were activated by 10 nM quercetin in L6 myotubes (Fig. 4A). A similar result was reported for adenosine, which increased glucose uptake via its A1 adenosine receptor (A1AR), instead of IR, in skeletal muscle cells^45^. The possible mechanism may involve a G protein-coupled receptor, which can participate in several processes and contribute to the activation of several response element-binding proteins and transcription factors, such as activation of PI3K/Akt signalling^46,47^. Indeed, a quercetin- and oleic acid-responsive G-protein-coupled receptor capable of modulating insulin secretion has been reported^48^. In addition, differential sensitivity of factors is likely to underlie why 1 nM quercetin activated PI3K phosphorylation without activating IRS1 and Akt phosphorylation^49^. This condition was similar to our previous research showing that propolis extract at 10–10^3^ ng/mL induced PI3K phosphorylation rather than aPKC phosphorylation, which is a downstream factor of PI3K^14^. Furthermore, PKCζ/λ was also phosphorylated by 10 nM quercetin (data not shown). Meanwhile, quercetin at a high concentration range (1 μM and 10 μM) also induced AMPK phosphorylation (Supplementary Fig. 2), consistent with previous results^38^.

In this study, quercetin at 0.1, 1, and 10 nM induced GLUT4 translocation in myotubes (Fig. 3A); nevertheless, only 0.1 nM and 1 nM quercetin increased glucose uptake (Fig. 2), indicating that GLUT4 translocation does not completely correlate with increased glucose uptake. Although GLUT4 is an essential factor for regulating glucose homeostasis, phosphorylation of p38 mitogen-activated protein kinase (MAPK) is required for function of GLUT4 after translocation to the plasma membrane to promote glucose uptake^50^. Indeed, inactivation of p38 MAPK decreased glucose uptake without affecting GLUT4 translocation^51^. Quercetin at 10 nM is likely to inactivate p38 MAPK phosphorylation. Furthermore, intracellular delivery of phosphatidylinositol (3,4,5)-trisphosphate is another possible factor, as previous research has demonstrated that PI(3,4,5)P_3_ ameliorates GLUT4 translocation to the plasma membrane without increasing glucose uptake^52^. Moreover, SOCS3, SHP1, SHP2, and IRS2 also reportedly promote GLUT4 translocation without increasing glucose uptake^53–55^. Therefore, some discrepancies between GLUT4 translocation and glucose uptake exist.

Our *in vivo* results revealed that quercetin at the physiological concentration range promoted GLUT4 translocation without altering the expression level of GLUT4 in muscle of mice 90 min after a single oral administration of EMIQ at 10 and 100 mg/kg body weight (Fig. 8B). Although EMIQ, which derives from rutin via enzymatic hydrolysis and contains a water soluble glucoside^56^, is not a natural product, it has recognized to be safe (Generally Recognized as Safe: GRAS) by U.S. Food and Drug Administration (U.S.FDA). It is reported that EMIQ was administrated up to 2.5% in diet (approximately 1600 mg/kg body weight/day) in 13-week repeated oral toxicity study in rats^57^. This result supported that the maximum dose of EMIQ used in this study is non-toxic. In addition, bioavailability of EMIQ is higher than that of natural quercetin glycosides^29,58^. Thus, we used this compound in this study. After intake quercetin glycosides, the intestinal mucosa is the mainly tissue to hydrolyze glycoside moiety and liberate aglycone form (quercetin)^28^. Formed quercetin is absorbed in small intestine and receive conjugation with glucuronate and/or sulfate or methylation. Isorhamnetin is one of mathylated form of quercetin. EMIQ is absorbed in mice and human as the same as quercetin glycosides from natural sources^29,59^. It has been reported that the plasma concentration of conjugated metabolites was increased and reached a maximal level at 90 min after intake of EMIQ in human^29^. Our results showed that a single oral administration of EMIQ at 10 and 100 mg/kg body weight promoted GLUT4 translocation and the concentrations of quercetin aglycone in the plasma were 4.95 ± 0.82 nM and 6.80 ± 2.00 nM, respectively. These results were fully consistent with our *in vitro* data using L6 myotubes, i.e., quercetin at 0.1–10 nM significantly promoted GLUT4 translocation (Fig. 3A). We could not detect isorhamnetin after administration of EMIQ at 10 and 100 mg/kg body weight, though conjugation form of isorhamnetin (Table 1). On the other hand, quercetin and its conjugated form were detected under the same conditions. Conjugation of quercetin is higher than that of isorhamnetin. These results suggest that methylation of quercetin in mice is slower than conjugation and formed isorhamnetin is rapidly received conjugation process in mice body.

In conclusion, our findings highlight the protective effects of quercetin and isorhamnetin at a physiological concentration range on glucose uptake in muscle cells. In addition, the mechanisms by which quercetin- and isorhamnetin-increased glucose uptake are different: quercetin principally activated CaMKKβ/AMPK and insulin signalling pathways, whereas isorhamnetin mainly activated the JAK/STAT pathway. Our findings reveal molecular mechanisms that support the use of quercetin and isorhamnetin as a novel therapeutic strategy for prevention and treatment of hyperglycaemia and associated disorders.

## Methods

### Materials

Quercetin, DMSO, acetonitrile, formic acid, and methanol were purchased from Wako Pure Chemical Industries (Osaka, Japan) and isorhamnetin was from Extrasynthese (Genay, France). AICAR, leptin, BAPTA-AM, resazurin, 2-deoxyglucose (2DG), glucose-6-phosphate dehydrogenase (G6PDH), sulfatase from *abalone entrailsand* (Type VIII), and β-glucuronidase from *Escherichia coli* (Type IX-A) were obtained from Sigma-Aldrich (St. Louis, MO). Diaphorase and ß-nicotinamide adenine dinucleotide phosphate (ß-NADP+) were from Oriental Yeast Co. Ltd. (Tokyo, Japan). Bovine serum albumin (BSA), Blocking-One and Blocking One-P solutions were from Nacalai Tesque (Kyoto, Japan). Polyvinylidene difluoride membrane was from GE Healthcare (Fairfield, WA, USA). Minimum essential medium (MEM) was from Nissui Pharmaceutical (Tokyo, Japan). Protease and phosphatase inhibitor cocktails were purchased from Roche Diagnostics (Tokyo, Japan). Lipofectamine™ RNAiMAX was purchased from Invitrogen Life Technologies (Burlington, Ontario, Canada). Reduced serum medium (Opti-MEM) and STO-609 were procured from Santa Cruz Biotechnology (Santa Cruz, CA. For western blotting analysis, anti-GLUT4 mouse IgG, anti-Akt rabbit IgG, anti-phospho-AMPKα (Thr 172) rabbit IgG, anti-AMPKα rabbit IgG, anti-JAK2 rabbit IgG, anti-phospho-JAK2 rabbit IgG, anti-STAT3 mouse IgG, anti-phospho-STAT3 (Tyr 705) rabbit mAb, anti-mouse IgG, and anti-rabbit IgG antibodies were from Cell Signaling Technology (Danvers, MA). Anti-STAT1, anti-STAT1 (phospho Y701), anti-STAT5a, and anti-STAT5a (phospho Y694) antibodies were from Abcam (Cambridge, MA). Anti-IRS1 rabbit IgG antibody was from Upstate Cell Signaling Solution (Lake Placid, NY). Anti-phospho-IRS1 (phospho Y896) and anti-phospho-tyrosine mouse mAb antibodies were from BD Transduction Laboratories (San Diego, CA). Anti-IR rabbit IgG, anti-ß-actin rabbit IgG, anti-phospho-Akt (Thr308) rabbit IgG and anti-phospho-Akt (Ser473) rabbit IgG were from sigma Chemical (St. Louis, MO). For AMPKα, JAK2, and JAK3 knockdown assay, the following siRNAs were used: 5′-GCA UAU GCU GCA GGU AGA-3′ and 5′-UCU ACC UGC AGC AUA UGC-3′ for AMPKα1 and AMPKα2, respectively; 5′-CCA CCC AAU CAU GUC UUC CAC AUA G-3′ and 5′-CUA UGU GGA AGA CAU GAU UGG GUG G-3′ for JAK2; 5′-GCU GGC AUU CUG GAC UGC AAG UAG A-3′ and 5′-UCU ACU UGC AGU CCA GAA UGC CAG C-3′ for JAK3 and 5′-AUU CUA UCA CUA GCG UGA CUU-3′ for the control.

### Cell culture and treatment

*In vitro* cultured cell experiments were conducted according to a previously described protocol^6,14^. Briefly, L6 myoblasts derived from rat skeletal muscle and of less than 40 passages were maintained in MEM supplemented with 10% foetal bovine serum (FBS) and antibiotics (100 U/ml penicillin and 100 μg/ml streptomycin) at 37 °C under a humidified atmosphere condition of 5% CO_2_ and 95% air. In each experiment, cells (2.2 × 10^4^ cells/ml) were seeded into 96-well plates or 60-mm dishes for induction of differentiation into mature myotubes. After reaching confluence, cells were supplemented with differentiation medium containing 2% FBS and the same antibiotics for 7 days. Cells were used for each experiment after morphological analysis of differentiation status using phase-contrast microscopy. To explore the contribution of CaMKKβ or intracellular-free calcium ions in quercetin- and isorhamnetin-stimulated GLUT4 translocation, L6 cells were incubated in the presence or absence of 10 μM STO-609 and 25 μM BADPA-AM, respectively, for 30 min prior to treatment with either quercetin or isorhamnetin.

### Animal treatment

All animal experiments were performed according to the guidelines for animal experiments at Kobe University Animal Experimentation Regulation and approved by the Institutional Animal Care and Use Committed of Kobe University (Permission 29-05-02). Male ICR mice (5 weeks old, n = 20) were obtained from Japan SLC (Shizuoka, Japan) and maintained at constant temperature (23 ± 2 °C) with a 12 h light-dark cycle (lights on at 8:00 am). Mice were subsequently randomly divided into four groups of five each, and acclimatized for 1 week with free access to tap water and a laboratory-purified diet (3.850 kcal/g diet) consisting of 76% carbohydrate, 15% protein and 9% fat) (Research Diets, Tokyo, Japan). For analysis of concentrations of quercetin and isorhamnetin in the plasma and GLUT4 translocation in the muscle, mice were orally administrated EMIQ at 10, 100, or 1000 mg/kg body weight, or water as a vehicle control after 18 h fasting. The mice were sacrificed 90 min after the administration under anesthesia using sodium pentobarbital and seroflurane, and euthanized by exsanguination from cardiac puncture. Plasma and soleus muscle were collected, and kept at -80 °C until use.

### Measurement of quercetin and isorhamnetin in the plasma of mice orally administrated EMIQ

To determine the levels of quercetin and isorhamnetin after administration of EMIQ, the obtained plasma were analyzed using HPLC (UV 370 nm) with or without deconjugation treatment using glucuronidase/sulfatase^16^. Briefly, an aliquot of 250 μl of plasma was mixed with 50 μl of 20% (w/v) ascorbic acid and incubated with 125 μl of 75 mM potassium phosphate buffer (pH 6.8) with or without 500 U/sample β-glucronidase and 125 μl of 200 mM sodium acetate buffer (pH 5.0) with or without 10 U/sample sulfatase for 2 h at 37 °C after adding 2 μl of 1 mM nobiletin as an internal standard. To separate quercetin and isorhamnetin, mixture was applied on a Sep-Pak C18 1 cc Vac Cartridge and eluted with 2 ml 95% (v/v) methanol. After eluate was dried by evaporation, dried material was dissolved in 50 μl of 50% (v/v) methanol and applied to HPLC. HPLC was performed using a SHIMADZU LabSolutions system (SHIMADZU, Kyoto, Japan) with SPD-M20A diode array detector. HPLC separation was done with a gradient system using 0.02% aqueous phosphoric acid as mobile phase A and acetonitrile as mobile phase B with a Cadenza CL-C18 column (φ 4.6 mm × 250 mm, Kyoto, Japan) at a flow rate of 1.0 ml/min. The gradient program was 0–13 min, 20% B, 13–33 min, 50–80% B, 33–43 min, 100% B, 43–60 min, 20% B.

### Glucose uptake assay

After serum starvation by incubating L6 myotubes in MEM with 0.2% BSA for 18 h, cells were treated with quercetin and isorhamnetin at the specified concentration (0.01 nM–10 μM) for 4 h in 0.2% BSA-containing medium. DMSO and insulin (100 nM) were used as a vehicle control and positive control, respectively. Glucose uptake was measured by an enzymatic 2DG uptake assay using myotubes on a 96-well plate as previously described^60^.

### Preparation of whole protein and plasma membrane fractions

For *in vitro* cell culture experiments, after serum starvation for 18 h, myotubes were treated with various concentrations of quercetin or isorhamnetin (0.1–10 nM) for 15 min; DMSO (final 0.1%) was used as a vehicle control. As positive controls, 100 nM insulin, 1 mM AICAR, 10 nM leptin, or 100 ng/mL IL-15 were used for insulin, AMPK, JAK2/STAT, or JAK3/STAT signalling pathways, respectively. Cells were treated with insulin or AICAR for 15 min, leptin for 60 min, or IL-15 for 24 h. Whole protein and plasma membrane fractions were prepared from myotubes, as previously described^61^. For *in vivo* experiment, plasma membrane fraction and tissue lysate of soleus muscle were prepared^61^.

### Western blot analysis

Translocation of GLUT4 to the plasma membrane, as well as expression and phosphorylation levels of GLUT4-related regulators were estimated by western blot analysis. Briefly, equal amounts of proteins were separated by sodium dodecyl sulfate-polyacrylamide gel electrophoresis (SDS-PAGE). Separated proteins were transferred onto a polyvinylidene fluoride membrane and nonspecific binding sites were blocked using either Blocking One (to detect unphosphorylated proteins) or Blocking One-P (to detect phosphoproteins). The membrane was incubated overnight with an appropriate primary antibody for GLUT4 (1:5000), IR (1:10000), p-IRS1 (1:5000), IRS1 (1:5000), p-PI3K (1:5000), PI3K (1:10000), p-Akt (1:5000), Akt (1:10000), p-AMPKα (1:5000), AMPKα (1:10000), p-ACC (1:5000), ACC (1:10000), p-LKB1 (1:5000), LKB1 (1:10000), p-JAK2 (1:5000), JAK2 (1:5000), p-JAK3 (1:5000), JAK3 (1:10000), p-STAT1 (1:5000), STAT1 (1:10000), p-STAT3 (1:5000), STAT3 (1:10000), p-STAT5 (1:5000), STAT5 (1:10000), or β-actin (1:20000), and subsequently treated with the corresponding horseradish peroxidase-conjugated secondary antibody (1:50000) for 1 h. Specific immune complexes were developed using ImmunoStar^®^ LD and detected with an ATTO Light-Capture II Western Blotting Detection System. Individual band density was calculated by ImageJ and normalized to the control.

### Immunoprecipitation

Aliquots of whole protein fractions (100 μg protein) were incubated with anti-IR antibody (1:100) in a rotator for 2 h at 4 °C. Next, 10 μl of protein A/G plus-agarose beads were added to the reaction mixture and incubated with rotation overnight at 4 °C. The pellet, which was combined with cell lysate, specific antibody, and agarose resin, was used for SDS-PAGE followed by immunoblot analysis.

### siRNA transfections

Mature myotubes were cultured in antibiotic-free medium and transfected with siRNA at a final concentration of 50 nM using Lipofectamine RNAiMAX transfection reagent according to the manufacturer’s instructions. In brief, mature myotubes were transfected with siRNA in Opti-MEM for 48 h. Subsequently, the medium was changed to fresh Opti-MEM and cells were treated with the indicated concentrations of quercetin or isorhamnetin.

### Statistical analysis

Data represent mean ± SD from at least three independent experiments. Statistical analysis was performed using Dunnett, Student’s *t* or Tukey–Kramer multiple-comparison tests. The statistical significance level was set at *p* < 0.05 using JMP 11.2.0.

## Supplementary information

Supplemental Dataset1
