# Supplementary material for: Quercetin and its metabolite isorhamnetin promote glucose uptake through different signalling pathways in myotubes
Source: Sci Rep. 2019 Feb 25;9:2690. doi: 10.1038/s41598-019-38711-7 (PMC6389993; doi:10.1038/s41598-019-38711-7)

Supplementary information

Quercetin and its metabolite isorhamnetin promote glucose uptake through different signalling pathways in myotubes

Hao Jiang[1], Yoko Yamashita[1], Asuka Nakamura[1], Kevin Croft[2], Hitoshi Ashida[1*]

Affiliations

[1]Department of Agrobioscience, Graduate School of Agricultural Science, Kobe University, Kobe, 657-8501, Japan.

[2] School of Biomedical Science, The University of Western Australia, Crawley, WA 6009, Australia.

***Corresponding author**

Hitoshi Ashida

Postal address: Graduate School of Agricultural Science, Kobe University, 1-1, Rokkodai-cho, Nada-ku, Kobe, Hyogo 657-8501, Japan. Tel: +81-78-803-5878.

E-mail: ashida@kobe-u.ac.jp.

Methods

Materials Quercetin and dimethyl sulfoxide (DMSO) were purchased from Wako Pure Chemical Industries (Osaka, Japan), while isorhamnetin was from Extrasynthese (Genay, Franch). 5-aminoimidazole-4-carboxyamide ribonucleoside (AICAR) and leptin were obtained from Sigma-Aldrich (St. Louis, MO). Bovine serum albumin (BSA), Blocking-One and Blocking One-P solutions were from Nacalai Tesque (Kyoto, Japan). Polyvinylidene difluoride membrane was from GE Healthcare (Fairfield, WA). Minimum essential medium (MEM) was from Nissui Pharmaceutical (Tokyo, Japan). Protease and phosphatase inhibitor cocktails were purchased from Roche Diagnostics (Tokyo, Japan). For western blotting analysis, anti-Akt rabbit IgG, anti-phospho-AMPKα (Thr 172) rabbit IgG, anti-AMPKα rabbit IgG, anti-JAK2 rabbit IgG, anti-phospho-JAK2 rabbit IgG, anti-mouse IgG, and anti-rabbit IgG antibodies were from Cell Signaling Technology (Danvers, MA). Anti-phospho-Akt (Thr308) rabbit IgG and anti-phospho-Akt (Ser473) rabbit IgG were from Sigma Chemical (St. Louis, MO).

Cell culture and treatment *In vitro* cultured cell experiments were conducted according to a previously described protocol[1,2]. Briefly, L6 myoblasts derived from rat skeletal muscle and of less than 40 passages were maintained in MEM supplemented with 10% FBS and antibiotics (100 U/mL penicillin and 100 μg/mL streptomycin) at 37ºC under a humidified atmosphere condition of 5% CO_2 and 95% air. In each experiment, cells (2.2×10^4 cells/mL) were seeded into 96-well plates or 60-mm dishes for induction of differentiation into mature myotubes. After reaching confluence, cells were supplemented with differentiation medium containing 2% FBS and the same antibiotics for 7 days. Cells were used for each experiment

after morphological analysis of differentiation status using phase-contrast microscopy.

Preparation of whole protein and plasma membrane fractions After serum starvation for 18 h, myotubes were treated with various concentrations of quercetin or isorhamnetin (1–10 μM) for 15 min; DMSO (final 0.1%) was used as a vehicle control. As positive controls, 100 nM insulin, 1 mM AICAR, or 10 nM leptin were used for insulin, AMPK or JAK2/STAT signalling pathways, respectively. Cells were treated with insulin or AICAR for 15 min, or leptin for 60 min. Whole protein and plasma membrane fractions were prepared from myotubes as previously described[3,4].

Western blot analysis Expression and phosphorylation levels of GLUT4-related regulators were estimated by western blot analysis. Briefly, equal amounts of proteins were separated by sodium dodecyl sulfate-polyacrylamide gel electrophoresis (SDS-PAGE). Separated proteins were transferred onto a polyvinylidene fluoride membrane and nonspecific binding sites were blocked using either Blocking One (to detect unphosphorylated proteins) or Blocking One-P (to detect phosphoproteins). The membrane was incubated overnight with an appropriate primary antibody for p-Akt (1:5000), Akt (1:10000), p-AMPKα (1:5000), AMPKα (1:10000), p-JAK2 (1:5000), or JAK2 (1:5000), and subsequently treated with the corresponding horseradish peroxidase-conjugated secondary antibody (1:50000) for 1 h. Specific immune complexes were developed using ImmunoStar® LD and detected with an ATTO Light-Capture II Western Blotting Detection System. Individual band density was calculated by ImageJ and normalized to the control.

All western blot data was performed the same gel and not combined from the different gels. In the supplementary Figure 4-12, the gel was cropped from parts showing red rectangle of

the same gel.

Statistical analysis Data are represented as mean ± SD from at least three independent experiments. Statistical analysis was performed using Dunnett or Tukey–Kramer multiple-comparison tests. The statistical significance level was set at $p < 0.05$ using JMP 11.2.0.

Results

Quercetin and isorhamnetin activated insulin-, AMPK- and JAK2/STAT-dependent pathways in L6 myotubes.

At higher concentrations (1 μM and 10 μM), quercetin and isorhamnetin significantly increased phosphorylation of Akt at Ser473 (Supplementary Fig. 1). At these concentrations, quercetin and isorhamnetin also induced phosphorylation of AMPK (Supplementary Fig. 2) and JAK2 (Supplemental Fig. 3). However, expression levels of these proteins was unaffected by treatment with these compounds.

References

1. Sawada, K., Yamashita, Y., Zhang, T., Nakagawa, K.& Ashida, H. Glabridin induces glucose uptake via the AMP-activated protein kinase pathway in muscle cells. *Mol Cell Endocrinol.* **5,** 99-108 (2014).

2. Ueda, M., Hayashibara, K. & Ashida, H. Propolis extract promotes translocation of glucase transporter 4 and glucose uptake through both PI3K- and AMPK-dependent pathways in skeletal muscle. *Biofactors.* **39,** 457-466 (2013).

3. Nishiumi, S. & Ashida, H. Rapid preparation of a plasma membrane fraction from adipocytes and muscle cells: application to detection of translocated glucose transporter 4 on the plasma membrane. *Biosci. Biotechnol. Biochem,* **71,** 2343-2346 (2007).

4. Yamamoto, N., Yamashita, Y., Yoshioka, Y., Nishiumi, S. & Ashida, H. Rapid preparation of a plasma membrane fraction: western blot detection of translocated glucose transporter 4 from plasma membrane of muscle and adipose cells and tissues. *Curr Protoc Protein Sci.* **85,** 1-12 (2016).

Figure legends

Figure S1. Effect of quercetin and isorhamnetin on insulin signalling in L6 myotubes. Differentiated L6 myotubes were treated with quercetin and isorhamnetin at the indicated concentrations for 15 min. Cell lysates were prepared and subjected to analysis of phosphorylation and expression of proteins in the insulin signalling pathway by western blotting. Arrow showed the target protein blots. Marked blots are presented in Supplementary Figure S10. Representative data are shown from independent triplicate analyses. Band density was measured and represented as the ratio of p-Akt to Akt. Values shown represent mean ± SD (n = 3). * and ** indicate significant differences from control cells by Dunnett's multiple comparison test (*p < 0.05 and**p< 0.01, respectively).

Figure S2. Effect of quercetin and isorhamnetin on the AMPK signalling pathway in L6 myotubes. Differentiated L6 myotubes were treated with quercetin and isorhamnetin at the indicated concentrations for 15 min. Cell lysates were prepared and subjected to analysis of phosphorylation and expression of proteins in the AMPK signalling pathway by western blotting. Arrow showed the target protein blots. Marked blots are presented in Supplementary Figure S11. Representative data are shown from independent triplicate analyses. Band density was measured and represented as the ratio of p-AMPK to AMPK. Values shown represent mean ± SD (n = 3). * and ** indicate significant differences from control cells by Dunnett's multiple comparison test (*p < 0.05 and**p < 0.01, respectively).

Figure S3. Effect of quercetin and isorhamnetin on the JAK/STAT signalling pathway in L6

myotubes. Differentiated L6 myotubes were treated with quercetin and isorhamnetin at the indicated concentrations for 15 min. Cell lysates were prepared and subjected to analysis of phosphorylation and expression of proteins in the JAK/STAT signalling pathway by western blotting. Arrow showed the target protein blots. Marked blots are presented in Supplementary Figure S12. Representative data are shown from independent triplicate analyses. Band density was measured and represented as the ratio of p-JAK2 to JAK2. Values shown represent mean ± SD ($n = 3$). * and ** indicate significant differences from control cells by Dunnett's multiple comparison test (*$p < 0.05$, and **$p < 0.01$, respectively).

Figure S4. This figure is supported information for Figure 3 in the manuscript.

Figure S5. This figure is supported information for Figure 4 in the manuscript.

Figure S6. This figure is supported information for Figure 5 in the manuscript.

Figure S7. This figure is supported information for Figure 6 in the manuscript.

Figure S8. This figure is supported information for Figure 7 in the manuscript.

Figure S9. This figure is supported information for Figure 8 in the manuscript.

Figure S10.This figure is supported information for Supplemental Figure S1 in the Supplemental data.

Figure S11. This figure is supported information for Supplemental Figure S2 in the Supplemental data.

Figure S12. This figure is supported information for Supplemental Figure S3 in the Supplemental data.

Supplemental Figure 1.

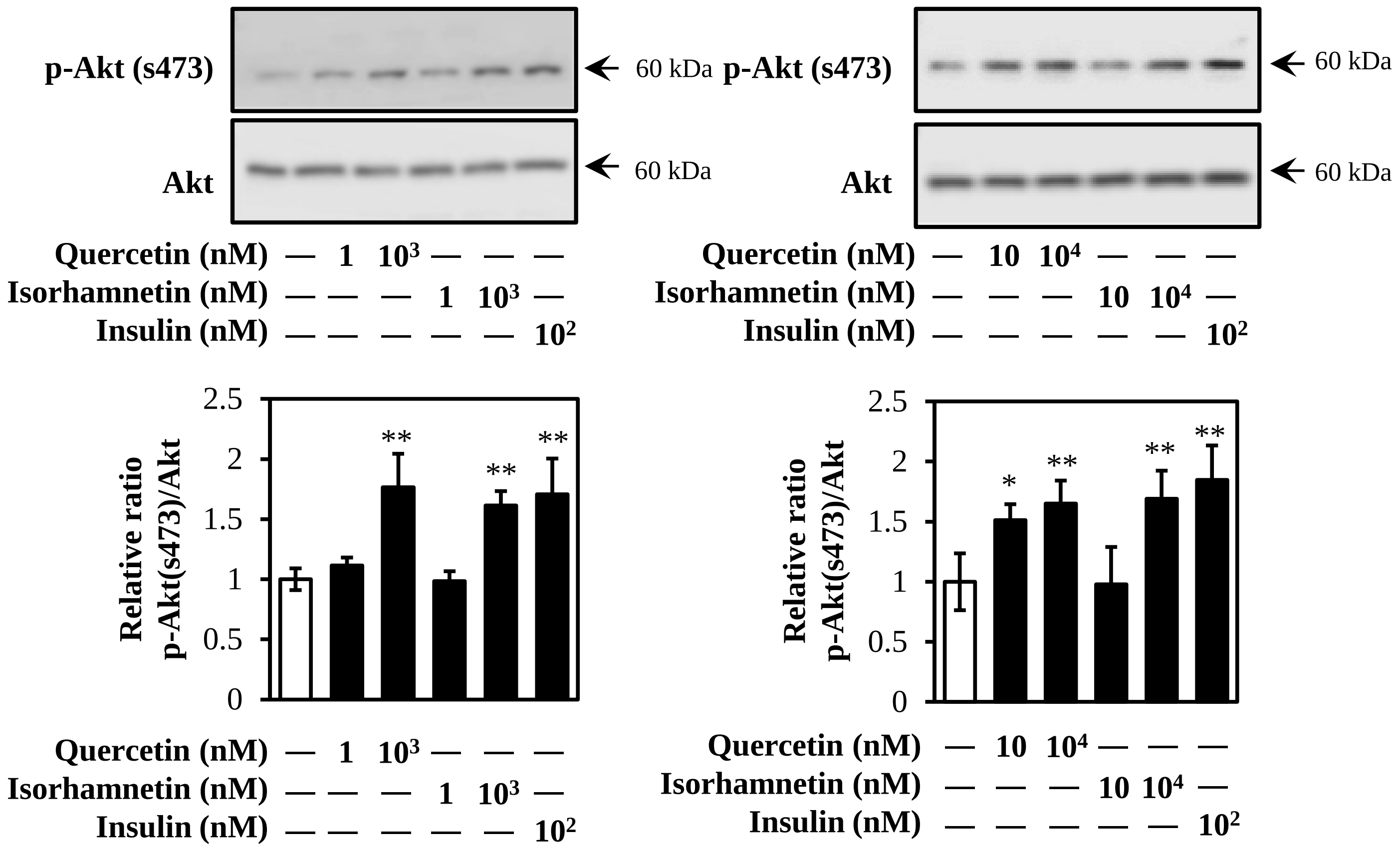

Supplemental Figure 2

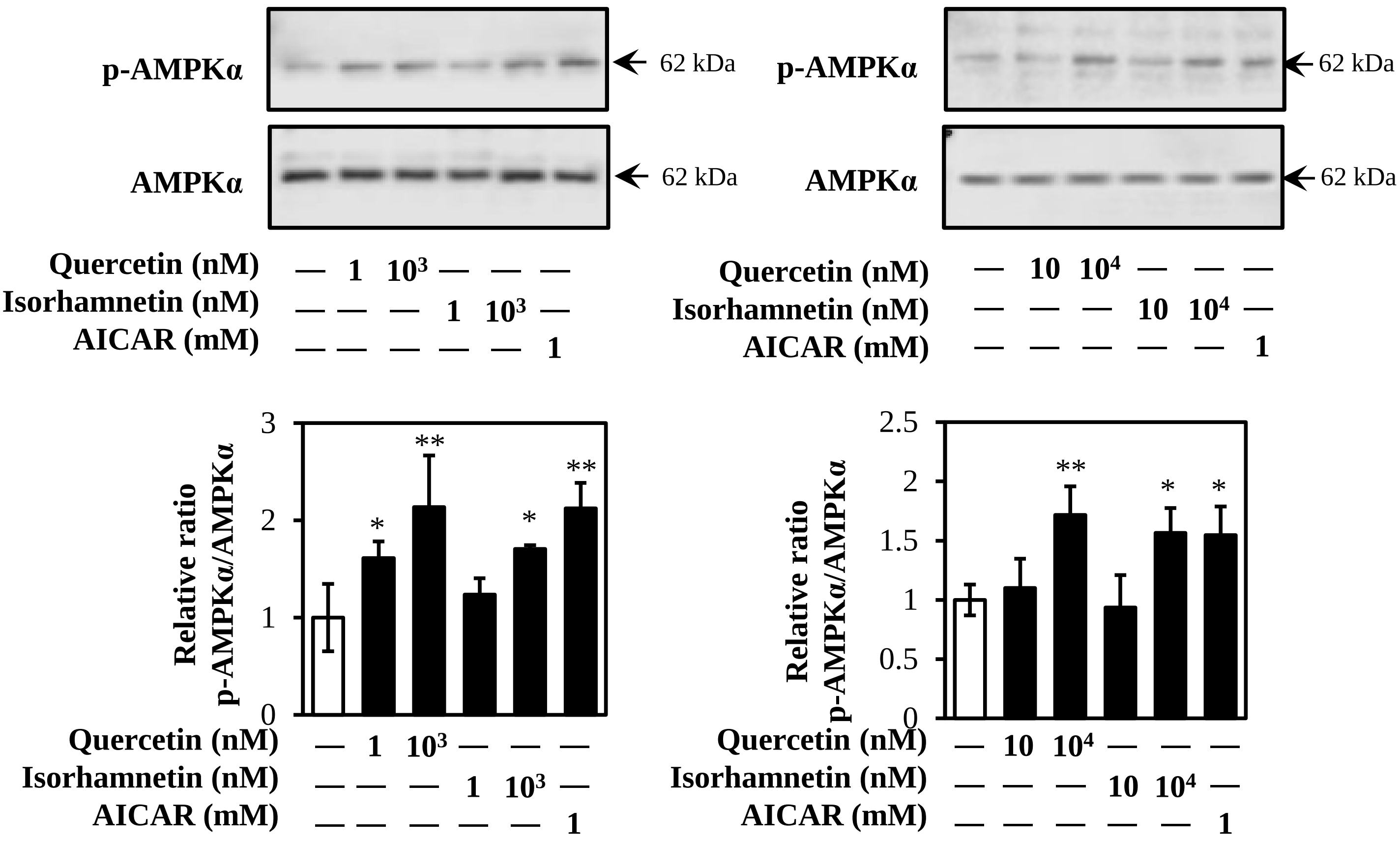

Supplemental Figure 3

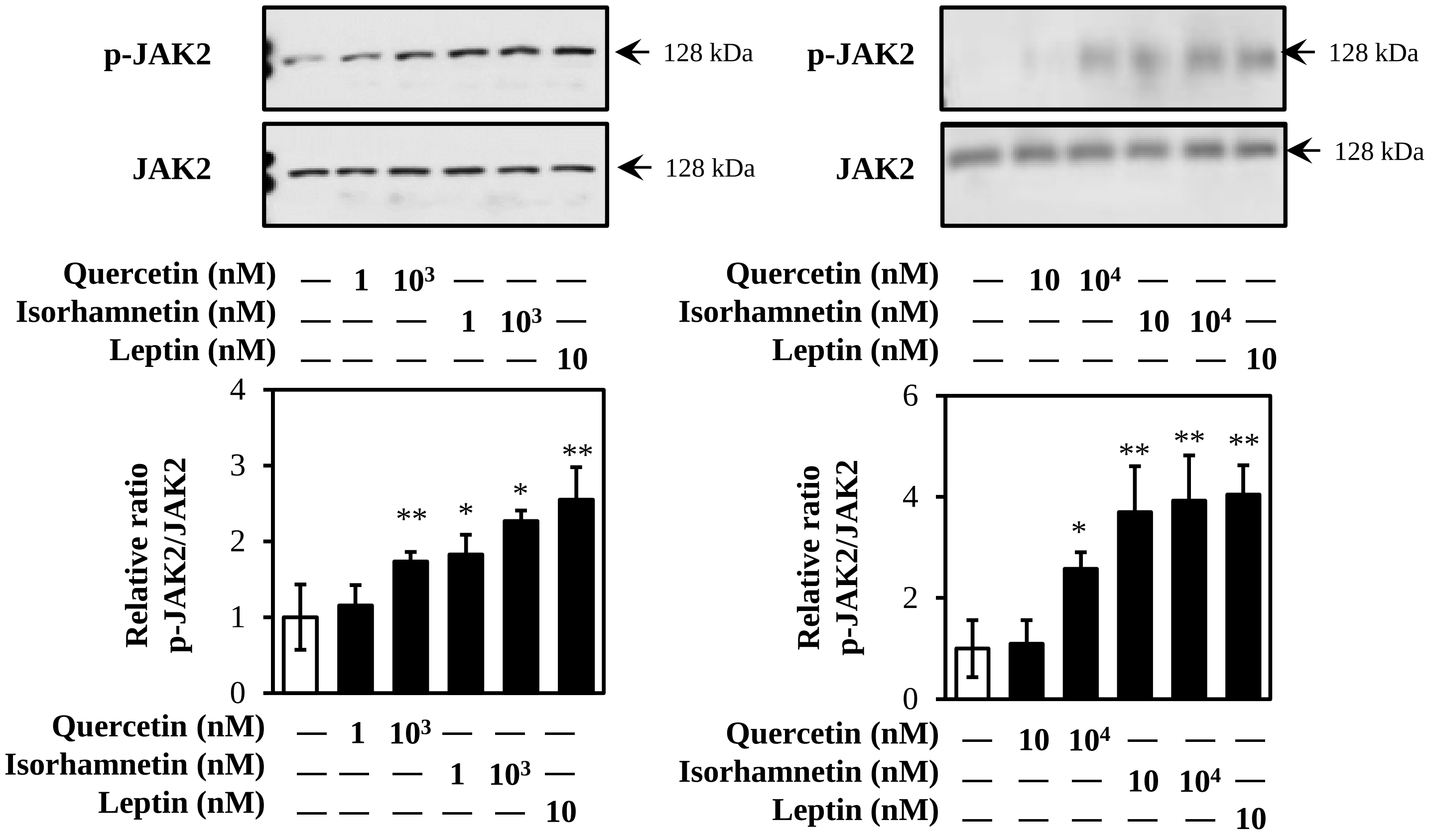

Supplemental Figure 4 (Support data for Manuscript Figure 3)

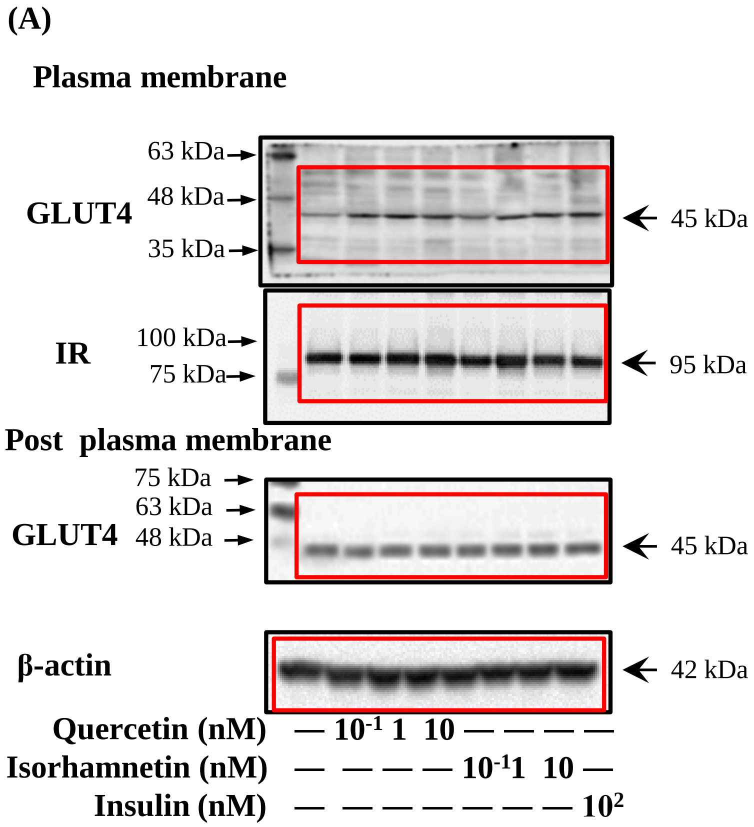

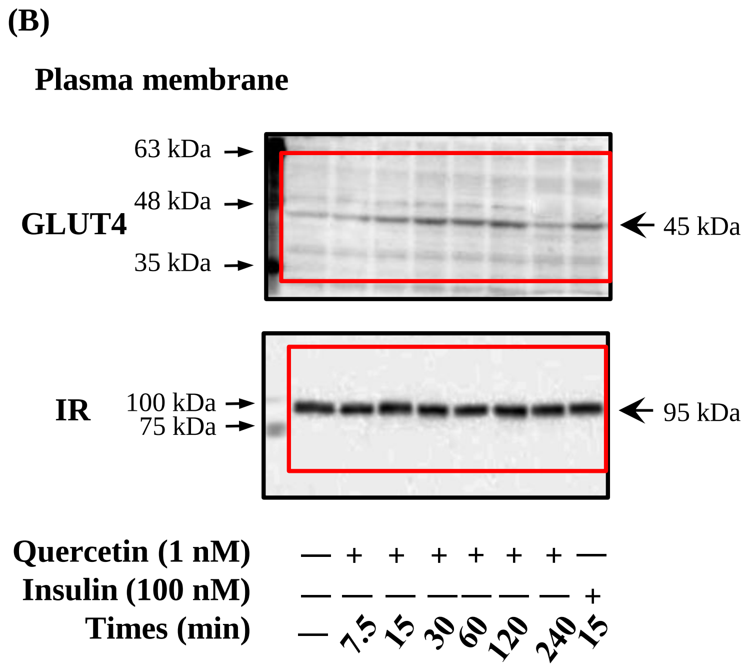

Supplemental Figure 5 (Support data for Manuscript Figure 4)

(A)

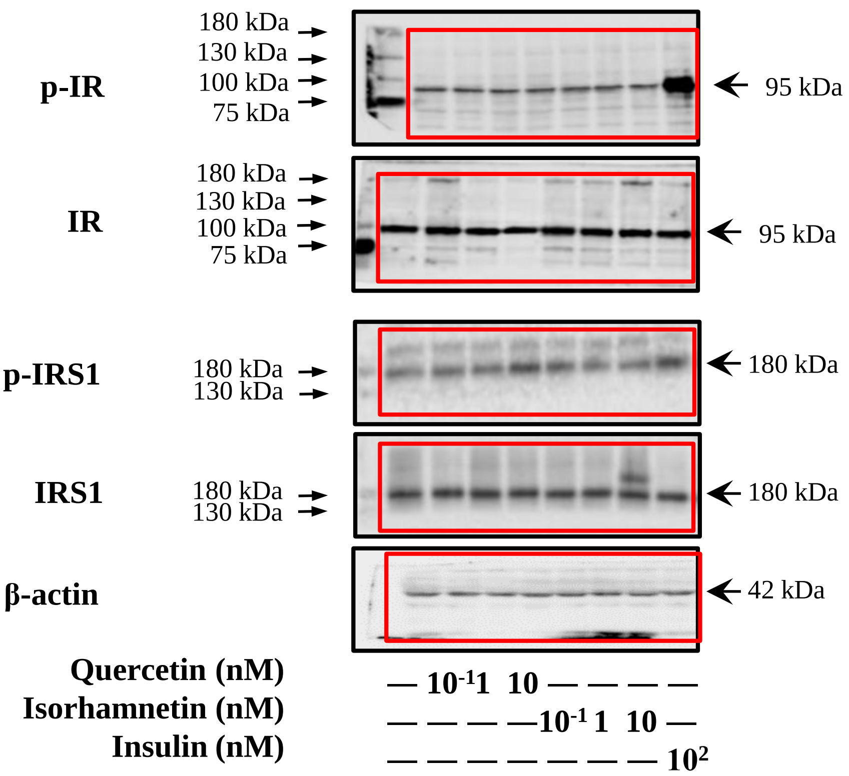

(B)

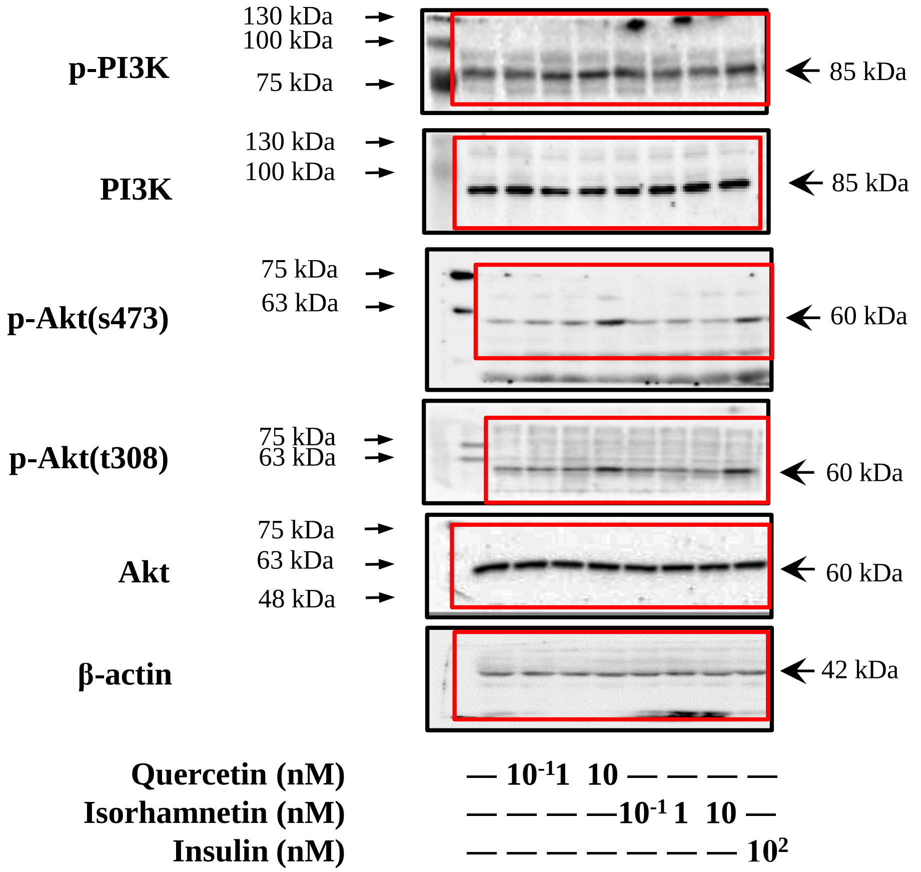

Supplemental Figure 6 (Support data for Manuscript Figure 5)

(A)

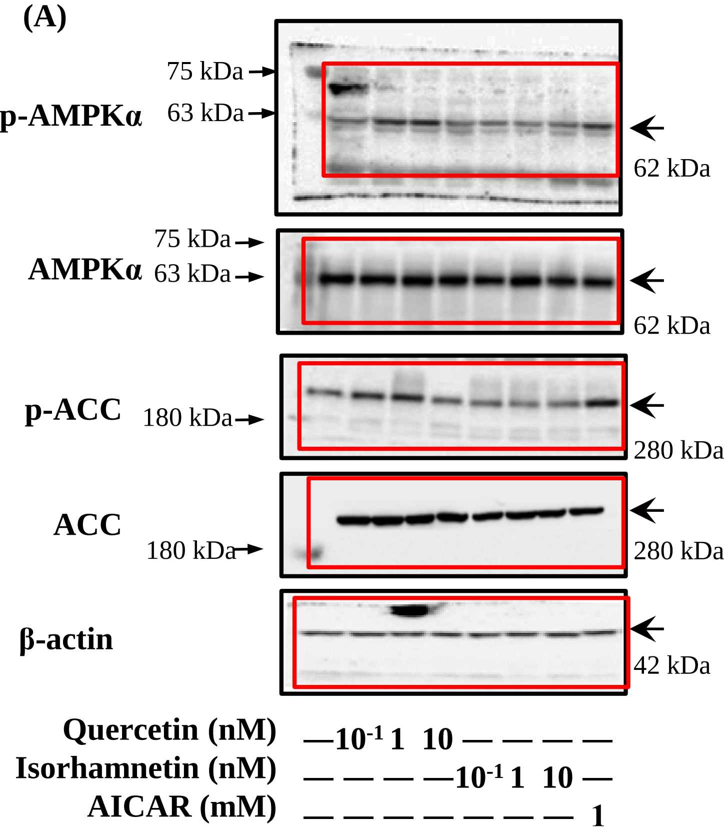

(B)

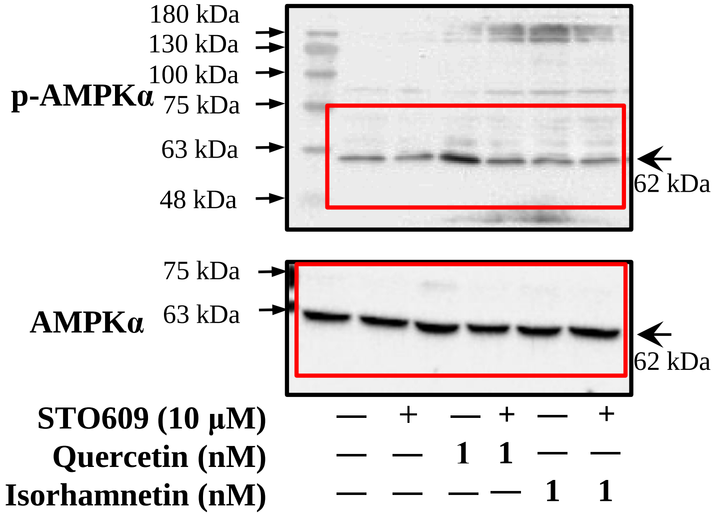

(C)

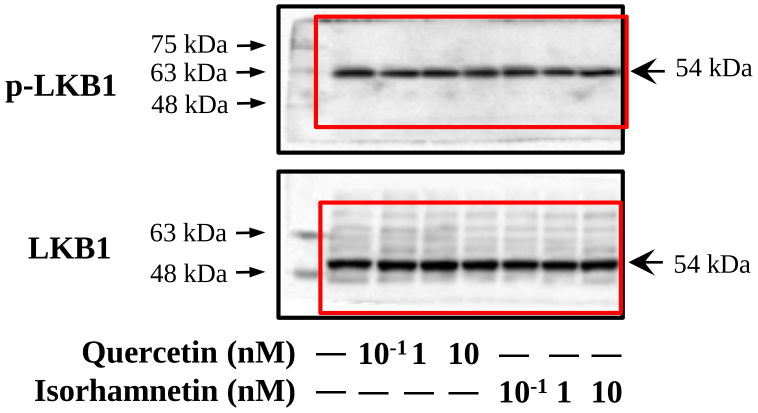

(D)

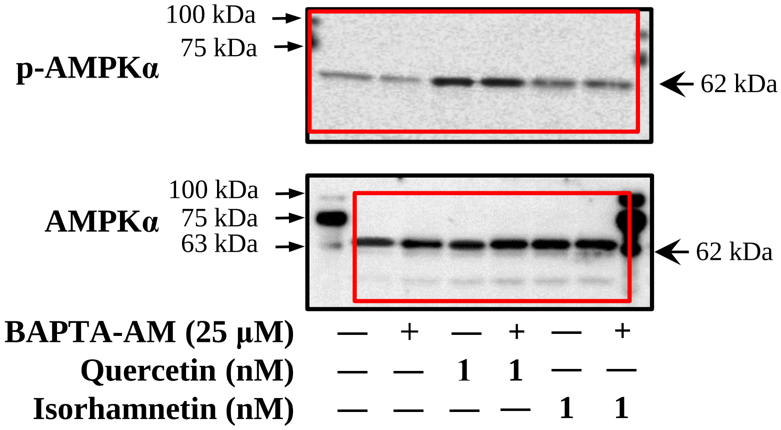

Supplemental Figure 7 (Support data for Manuscript Figure 6)

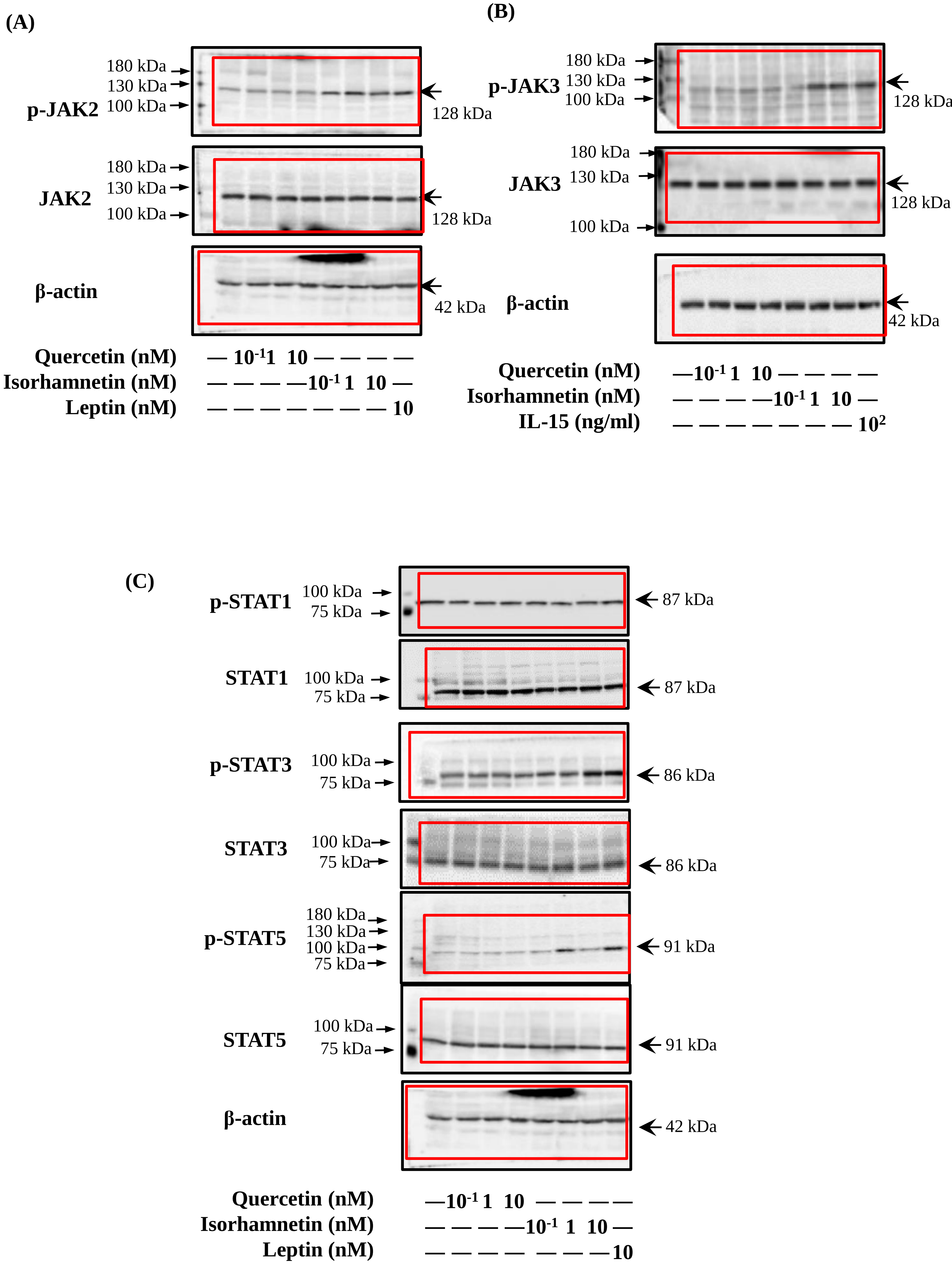

Supplemental Figure 8 (Support data for Manuscript Figure 7)

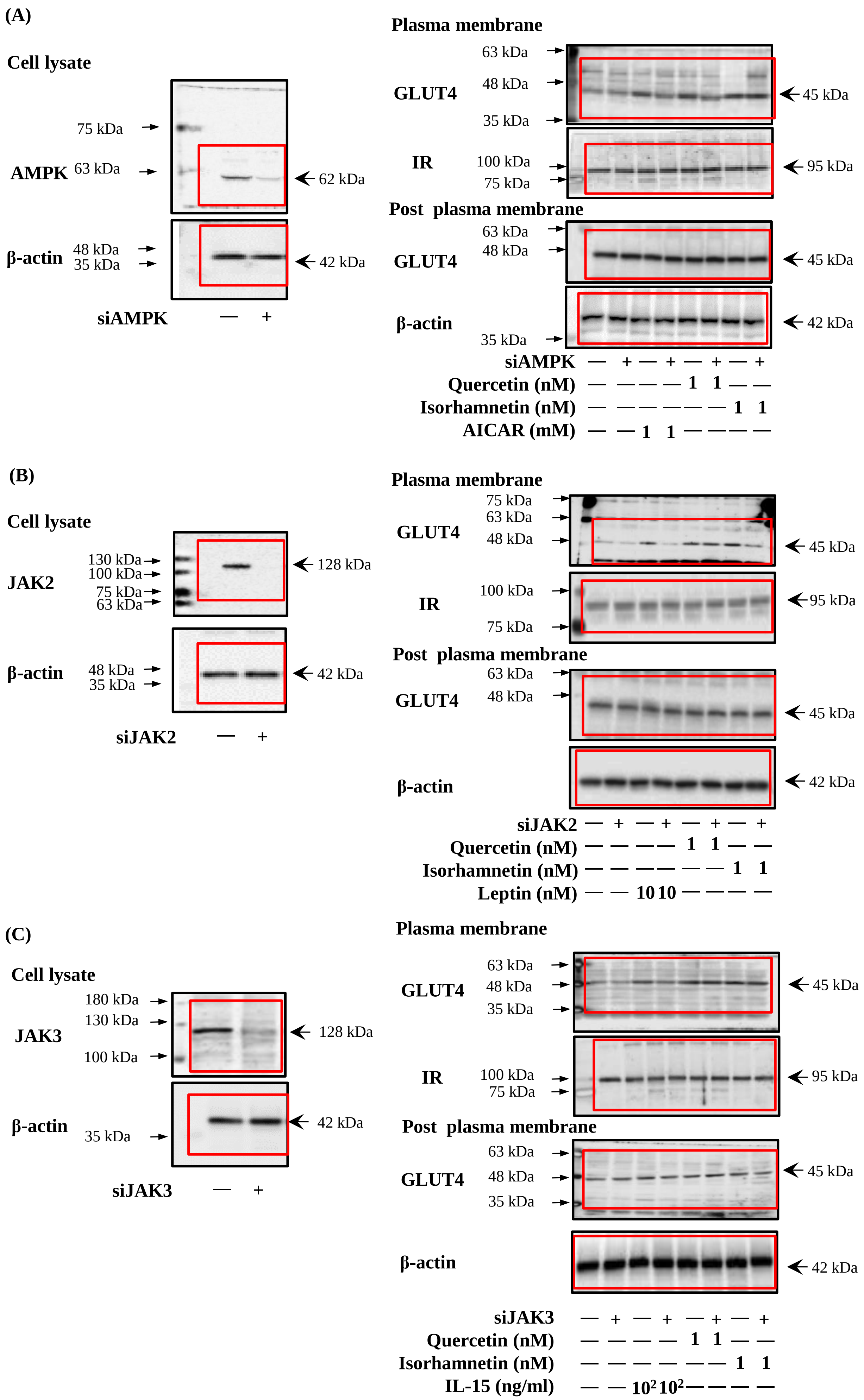

Supplemental Figure 9 (Support data for Manuscript Figure 8)

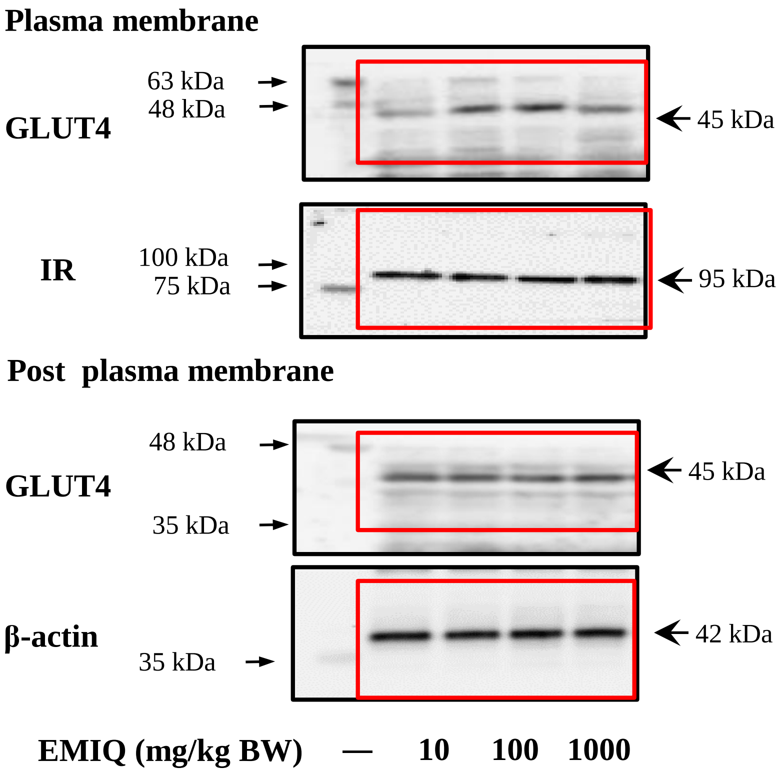

Supplemental Figure 10 (Support data for Supplemental Figure 1)

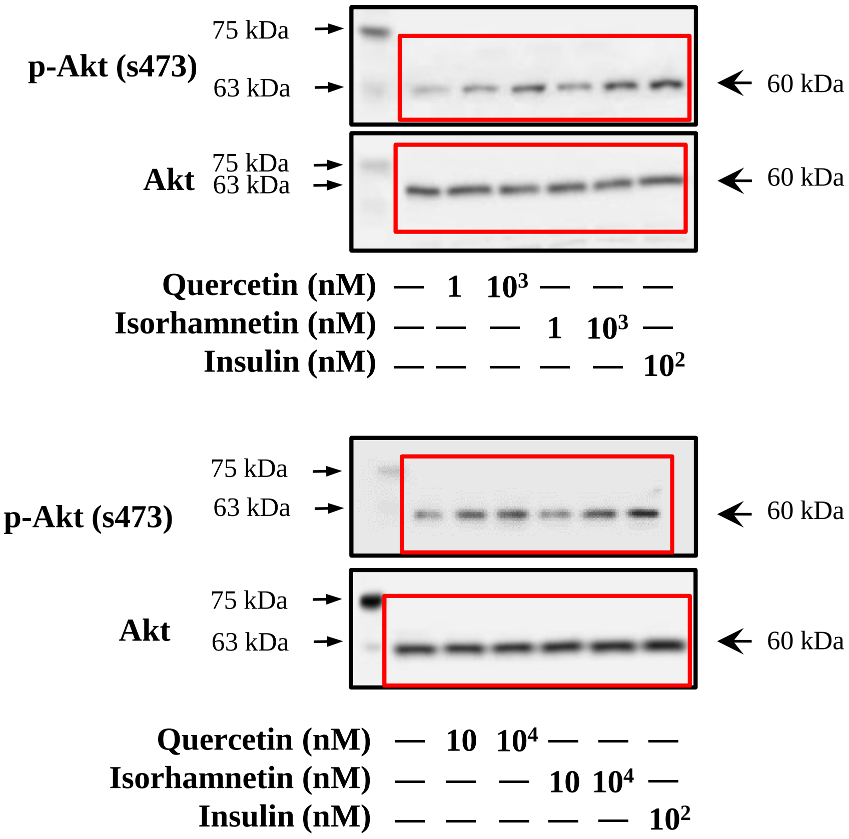

Supplemental Figure 11 (Support data for Supplemental Figure 2)

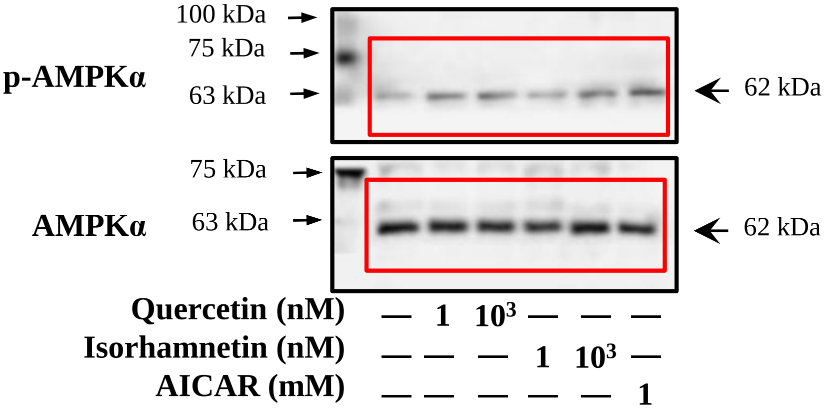

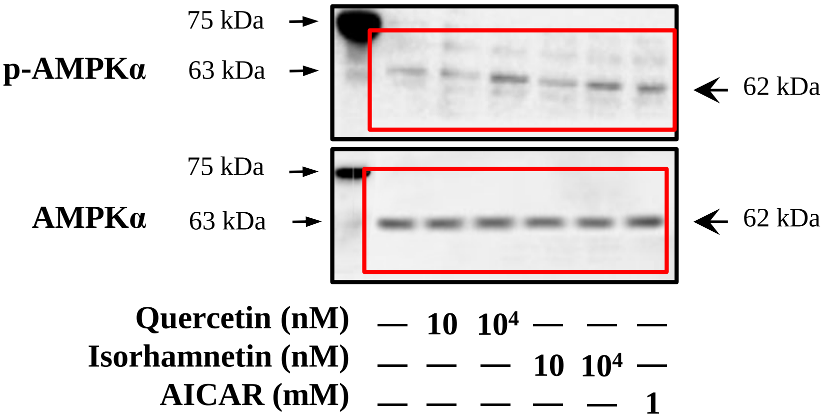

Supplemental Figure 12 (Support data for Supplemental Figure 3)

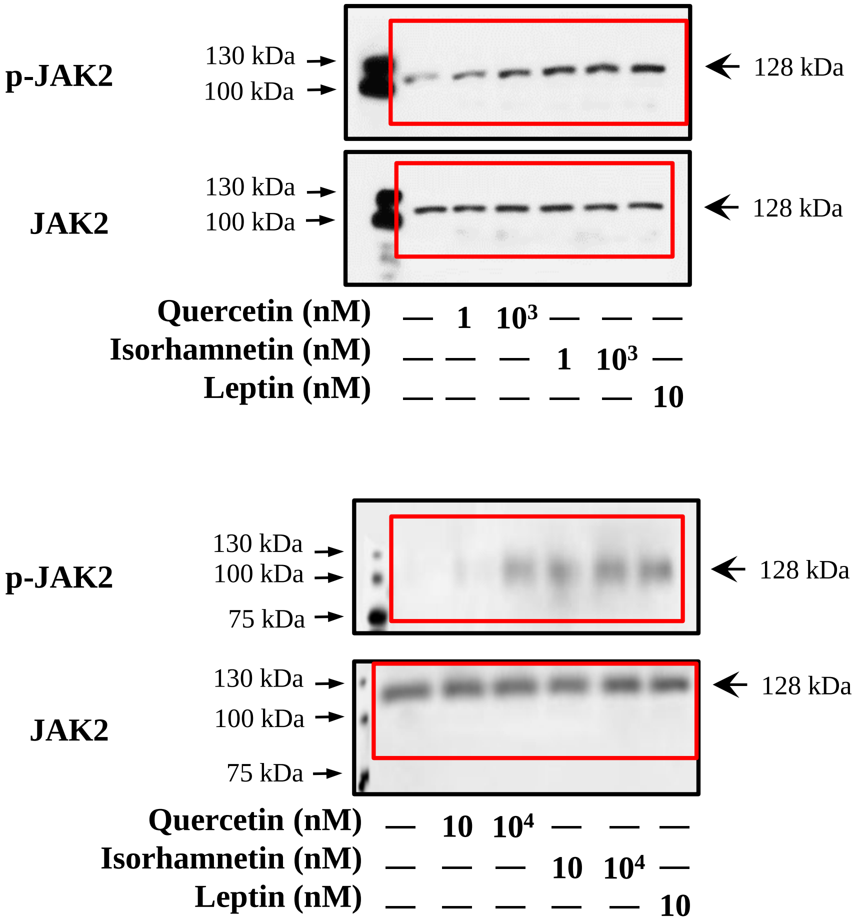

Supplement: Supplementary file 1 — Supplemental Dataset1 [file 41598_2019_38711_MOESM1_ESM.pdf]
